# Inhibitors identify an auxiliary role for mTOR signalling in necroptosis execution downstream of MLKL activation

**DOI:** 10.1042/BCJ20240255

**Published:** 2024-08-27

**Authors:** Sarah E. Garnish, Christopher R. Horne, Yanxiang Meng, Samuel N. Young, Annette V. Jacobsen, Joanne M. Hildebrand, James M. Murphy

**Affiliations:** 1 Walter and Eliza Hall Institute of Medical Research, 1G Royal Parade, Parkville, Victoria 3052, Australia; 2 Department of Medical Biology, University of Melbourne, Parkville, Victoria 3052, Australia; 3 Drug Discovery Biology, Monash Institute of Pharmaceutical Sciences, Monash University, Parkville, Victoria 3052, Australia

**Keywords:** mechanistic target of rapamycin, necroptosis, protein kinase, pseudokinase, signalling

## Abstract

Necroptosis is a lytic and pro-inflammatory form of programmed cell death executed by the terminal effector, the MLKL (mixed lineage kinase domain-like) pseudokinase. Downstream of death and Toll-like receptor stimulation, MLKL is trafficked to the plasma membrane via the Golgi-, actin- and microtubule-machinery, where activated MLKL accumulates until a critical lytic threshold is exceeded and cell death ensues. Mechanistically, MLKL's lytic function relies on disengagement of the N-terminal membrane-permeabilising four-helix bundle domain from the central autoinhibitory brace helix: a process that can be experimentally mimicked by introducing the R30E MLKL mutation to induce stimulus-independent cell death. Here, we screened a library of 429 kinase inhibitors for their capacity to block R30E MLKL-mediated cell death, to identify co-effectors in the terminal steps of necroptotic signalling. We identified 13 compounds — ABT-578, AR-A014418, AZD1480, AZD5363, Idelalisib, Ipatasertib, LJI308, PHA-793887, Rapamycin, Ridaforolimus, SMI-4a, Temsirolimus and Tideglusib — each of which inhibits mammalian target of rapamycin (mTOR) signalling or regulators thereof, and blocked constitutive cell death executed by R30E MLKL. Our study implicates mTOR signalling as an auxiliary factor in promoting the transport of activated MLKL oligomers to the plasma membrane, where they accumulate into hotspots that permeabilise the lipid bilayer to cause cell death.

## Introduction

Necroptosis is caspase-independent form of programmed cell death characterised by cell swelling, lysis and expulsion of cellular contents into the extracellular milieu. The expulsion of damage-associated molecular patterns (DAMPs) is known to elicit an immune response [1], which can trigger inflammation. Consequently, necroptosis is considered to have arisen as an altruistic cell death mode to counter pathogens [2–6]. However, much of the recent interest in this pathway has arisen from the observation that dysregulated necroptosis occurs in many human diseases [7–17], and the deletion of key pathway effectors can protect mice from disease in experimental models [18–25]. Because the terminal pathway effectors are dispensable for development [23,26–28], the necroptotic cell death pathway has attracted substantive interest as pharmacological targets to counter inflammatory diseases.

Necroptosis relies on three core proteins: the kinases, receptor-interacting serine/threonine protein kinase (RIPK)-1 and RIPK3; and the terminal effector, the mixed lineage kinase domain-like (MLKL) pseudokinase [29,30]. Necroptotic signalling is initiated upon ligation of the TNF or other death receptors [31,32], pathogen sensors, such as Toll-like receptors (TLR)-3 and TLR-4 [33] and the intracellular pathogen RNA sensor, ZBP1 [34,35]. In contexts where the proteolytic caspase enzymes and the E3 Ubiquitin ligases of the cIAP (cellular inhibitor of apoptosis proteins) family are depleted or disarmed, necroptosis ensues. MLKL resides in a dormant form bound to its upstream regulator, RIPK3 under basal conditions [36,37]. Upon the triggering of necroptosis, the RIPK3:MLKL subcomplex is recruited to a high molecular mass cytoplasmic platform termed the necrosome, which is nucleated by oligomeric assembly of the RIPK1 and RIPK3 kinases via functional amyloids formed by their RHIM (receptor homotypic interaction motif) sequences [38,39]. Here, RIPK3 phosphorylates MLKL to instigate three key events: (1) MLKL dissociation from the necrosome [40]; (2) a co-ordinate conformational interconversion of MLKL's pseudokinase domain [40]; and (3) MLKL assembling into oligomers that are then trafficked by the Golgi-, actin- and microtubule-machinery to the plasma membrane [41]. Activated MLKL oligomers accumulate in hotspots at the plasma membrane until a critical threshold is met to enable MLKL to permeabilise the lipid bilayer and induce cell death [41].

Despite extensive study of the terminal events mediated by MLKL over the past decade, there are several important gaps in knowledge. Thus far, the molecular cues directing the recruitment of the RIPK3:MLKL subcomplex to the necrosome, the stoichiometry of MLKL oligomers, co-effectors in trafficking MLKL oligomers to the plasma membrane, whether co-effector proteins are required for membrane permeabilisation, and the precise mechanism of MLKL-mediated membrane permeabilisation remain poorly understood. Targeted studies and screens have implicated ESCRT-trafficking, extracellular vesicle formation and inhibitory phosphorylation events in MLKL in negating necroptotic signalling [8,42-45], yet few studies have identified co-effector proteins that promote necroptosis. To date, CK1 and TAM kinases, RGMb, Thioredoxin-1 have been proposed to serve auxiliary functions in promoting necroptotic signalling [46-49], although whether these functions are context-dependent and more broadly conserved are currently unclear. The identities of other co-effector proteins are currently unknown and remain of outstanding interest.

Previous screens have employed small molecule drug libraries to identify candidate downstream co-effector proteins based their ability to inhibit cell death mediated by an activated MLKL mutant. These studies have pinpointed a role for the kinase co-chaperones, HSP90:Cdc37, in promoting necroptosis [50], consistent with the requisite function of the HSP90:Cdc37 co-chaperones in directing the folding of kinases and pseudokinases, including RIPK1, RIPK3 and MLKL [51-55]; and for RIPK1 as serving a role downstream of MLKL in necroptosis signalling [56,57]. These screens relied on activated mouse MLKL mutants, which unlike human MLKL [58], were found to be readily activated by mutations in the pseudokinase domain that mimic the molecular switch mechanism [26]. Because human MLKL signalling is more tightly regulated than the mouse orthologue, only recently has a human MLKL mutant, R30E, been identified as an activated mutant that signals for death constitutively on induction of expression and in the absence of necroptotic stimuli [59]. Here, we used human R30E MLKL expressing cells to screen a library of 429 kinase inhibitors for their capacity to inhibit cell death. We identified 13 inhibitors, with known targets in the mammalian target of rapamycin (mTOR) signalling pathway, which overall blocked cell death by preventing MLKL translocation to the plasma membrane. Based on these findings, we propose that inhibiting mTOR signalling impacts vesicular transport by the Golgi and actin machinery, which in turn prevents MLKL trafficking and accumulation at the plasma membrane hotspots required to execute necroptotic cell death.

## Results

### R30E MLKL-induced necroptotic cell death relies on unknown kinases

Previously, we reported R30E MLKL executed constitutive cell death upon expression in *MLKL^−/−^* HT29 cells [59]. R30E is an artificial mutation in human MLKL that disrupts the salt bridge between the four-helix bundle (4HB) domain (R30) and the brace region (E136 and D140) by the introduction of a negative charge. While substitutions at R30 of MLKL have not been reported in ClinVar or the Catalogue of Somatic Mutations in Cancer (COSMIC) [60,61], the R30E mutation was selected as an experimental tool to dissect mechanisms of downstream MLKL signalling. Specifically, R30E MLKL expression in cells leads to the formation of high-order MLKL oligomers that associate with biological membranes and enact cell death without additional stimulation [59]. We previously observed that constitutive cell death by R30E MLKL was partially inhibited by RIPK3 inhibitor, GSK′872 [59]. We attributed this inhibition to an essential upstream role of RIPK3 autophosphorylation in facilitating the assembly of the basal RIPK3:MLKL subcomplexes that are subsequently recruited to necrosomes and connected to the trafficking machinery following induction of necroptosis.

**Figure 1.**
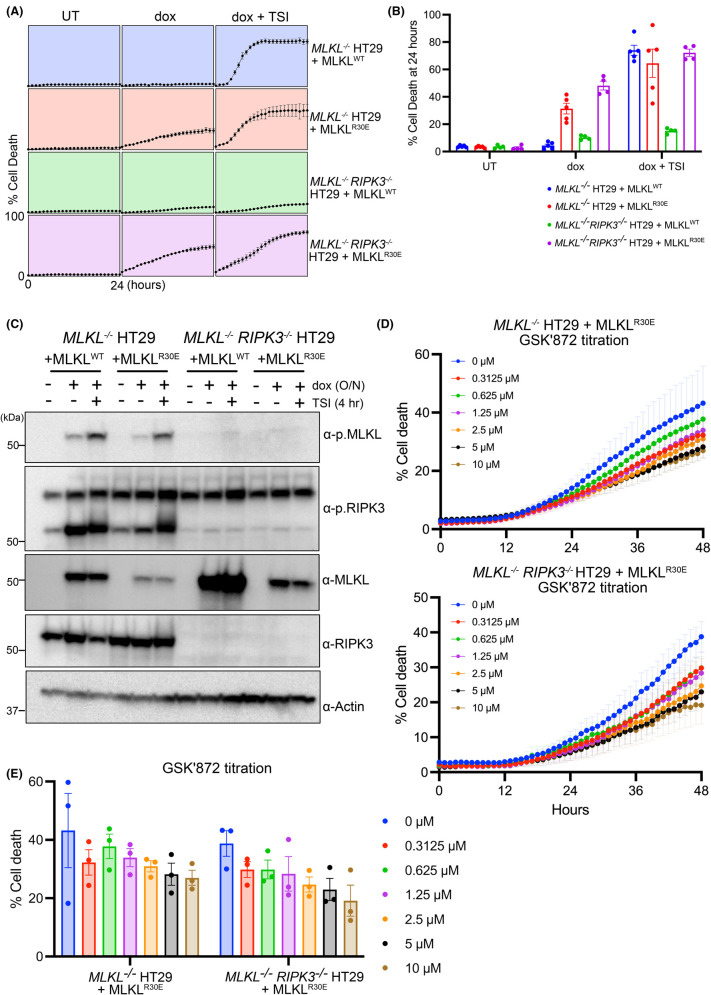
RIPK3 deletion does not inhibit R30E MLKL-mediated cell death. (**A**,**B**) Evaluation of necroptotic signalling by wild-type and R30E full-length human MLKL in *MLKL^−/−^* and *MLKL^−/−^ RIPK3^−/−^* HT29 cells. Human MLKL expression was induced with doxycycline (Dox; 100 ng/ml) and cell death was measured in the presence or absence of a necroptotic stimulus (TNF, Smac mimetic, IDN-6556; TSI). Cell death was quantified as a percentage of SYTOX Green-positive cells using IncuCyte SX5 live cell imaging. *MLKL^−/−^ RIPK3^−/−^* HT29 independent lines were assayed in *n = *2 for each of two independent cell lines for a total of *n = *4 independent experiments. One *MLKL^−/−^* HT29 cell line was assayed in *n = *2 and the other in *n = *3, for a total of *n = *5 independent experiments. Data are plotted as mean ± SEM. (**C**) *MLKL^−/−^* or *MLKL^−/−^ RIPK3^−/−^* HT29 cells were stimulated with doxycycline (Dox; 100 ng/ml overnight) to induce the expression of wild-type or R30E human MLKL. Protein levels of MLKL and RIPK3, both unphosphorylated and phosphorylated forms, were detected by immunoblotting in the presence or absence of necroptotic stimulation (TSI; 4 h). Data are representative of duplicate independent experiments. (**D**,**E**) *MLKL^−/−^* and *MLKL^−/−^ RIPK3^−/−^* HT29 cells stably transduced with *MLKL^R30E^* were treated with doxycycline (Dox) in the presence of RIPK3 kinase inhibitor, GSK′872, at increasing concentrations. IncuCyte SX5 imaging was used to quantify the percentage of cell death (SYTOX Green-positive cells) every hour for 48 h. Data are plotted as mean ± SEM of *n = *3. One independent cell line transduced with *MLKL^R30E^* was assayed in *n = *3 independent experiments.

To further investigate the relationship of R30E MLKL-mediated cell death to RIPK3, we expressed R30E MLKL in *MLKL^−/−^ RIPK3^−/−^* HT29 cells and tested the ability of R30E MLKL to signal for cell death. Wild-type (WT) and R30E MLKL exogene expression was induced with doxycycline, and the capacity to undergo cell death was assessed in the absence or presence of the necroptotic stimulus (TSI: T, TNF; S, Smac mimetic Compound A; I, pan-Caspase inhibitor IDN-6556/Emriscasan) by monitoring SYTOX Green uptake using IncuCyte live cell imaging. Unexpectedly, deletion of *RIPK3* did not inhibit R30E MLKL-induced cell death (Figure 1A,B), which contrasts the RIPK3 inhibitor, GSK′872, blocking R30E MLKL-mediated death in *MLKL^−/−^* HT29 cells [59]. Importantly, cell death occurred independently of RIPK3-mediated phosphorylation of MLKL (Figure 1C), consistent with our earlier finding [59] and indicative of a mode of MLKL activation distinct from RIPK3-mediated phosphorylation triggering MLKL release from the necrosome, a change in conformation and oligomerization. We next assessed if GSK′872 inhibited R30E MLKL-mediated death in a concentration-dependent manner (Figure 1D,E, Supplementary Figure S1A,B). *MLKL^−/−^* HT29 cells that were stimulated with doxycycline to induce expression of R30E MLKL were inhibited by concurrent treatment with GSK′872 in a concentration-dependent manner (Figure 1D). GSK′872 suppressed R30E MLKL-mediated death in *MLKL^−/−^ RIPK3^−/−^* HT29 cells to a similar extent as observed in *MLKL^−/−^*HT29 cells (Figure 1E). This is suggestive that GSK′872 protects cells from death through a mechanism that is not dependent on RIPK3.

GSK′872 was reported to inhibit 20 human protein kinases when assessed at 1 µM against a panel of 301 protein kinases [62]. GSK′872 exhibited 70–90% inhibition for 11 of these kinases and 50–70% inhibition of a further nine kinases [62]. Considering the dispensability of RIPK3 for R30E MLKL-mediated death, our findings raise the possibility that GSK′872 inhibition of R30E MLKL killing arises from off-target inhibition of other kinases, which prompted us to further explore whether additional kinases contribute to necroptotic signalling.

### Screening for kinase inhibitors that counter R30E MLKL constitutive death

**Figure 2.**
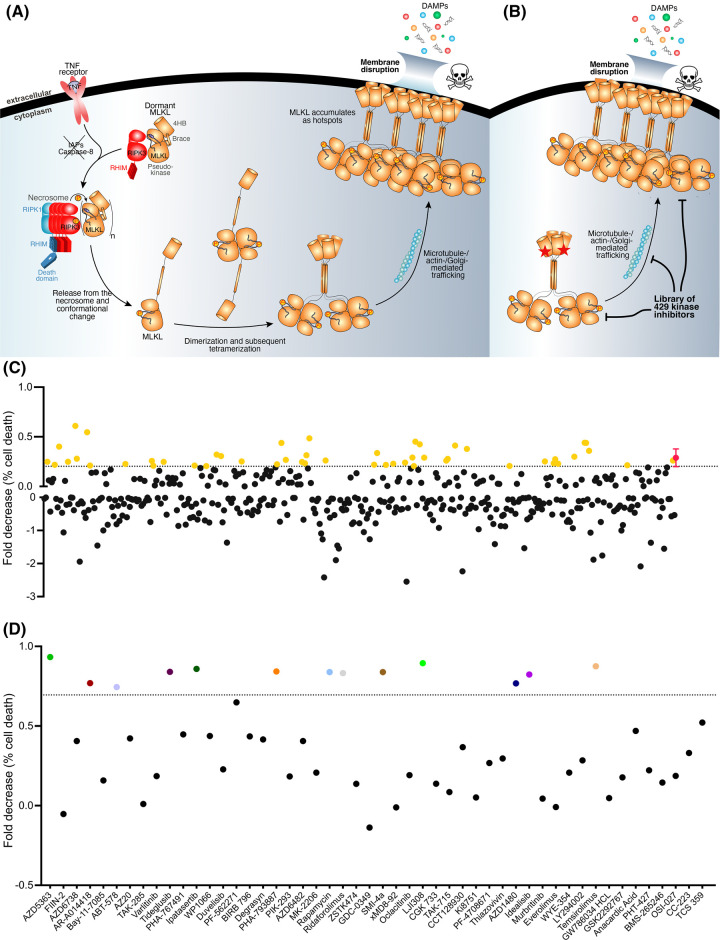
Screen identifies 13 kinase inhibitors that block R30E MLKL-mediated cell death. (**A**) Schematic of the necroptosis pathway. TNF (T) stimulates the TNFR1; cIAP1/2 activity is blocked with Smac mimetic (S); and the pan-caspase inhibitor, IDN-6556 (I), blocks caspase-8 activity. This leads to the formation of the necrosome, subsequent phosphorylation and activation of MLKL. Activated MLKL oligomerizes and traffics to biological membranes where its accumulation facilitates membrane permeabilization. (**B**) The activated R30E MLKL mutant (highlighted by red star) oligomerizes and initiates cell death in the absence of TSI stimulation or RIPK3 activation enabling a screen for inhibitors of necroptosis downstream of MLKL activation. The skull and crossbones image (Mycomorphbox_Deadly.png; by Sven Manguard) is used via a Creative Commons Attribution-Share Alike 4.0 license. (**C**) Phenotypic screen of 429 kinase inhibitors to identify rescue of R30E MLKL constitutive cell death. *MLKL^−/−^* HT29 cells were stimulated with 100 ng/ml doxycycline to induce expression of R30E MLKL. Following 16 h of induction, cells were treated with 1 µM compound. IncuCyte SX5 imaging was used to quantify the percentage of cell death by determining the number SYTOX Green-positive cells (dead cells) relative to the number of DRAQ5-positive cells (total cell number) at 24 h post-stimulation. Mean of technical duplicates for each compound is plotted as fold decrease in percentage cell death. GSK′872 depicted in pink, with mean* *±* *SD of *n *= 11 independent biological replicates. Positive hits that displayed a fold decrease in R30E MLKL-mediated cell death ≥0.2027 (dotted line; GSK′872 — 1 SD) are depicted in yellow. (**D**) Fifty positive hit compounds were re-screened for their capacity to inhibit R30E MLKL cell death. *MLKL^−/−^* HT29 cells were stimulated with 100 ng/ml doxycycline to induce expression of R30E MLKL and stimulated with 1 µM of compound simultaneously. IncuCyte SX5 imaging was used to quantify the percentage of cell death (SYTOX Green-positive cells) at 24 h post-stimulation. Data plotted as fold decrease between R30E MLKL-mediated cell death in the absence and presence of the compound. Plotted data represent one independent replicate. Dotted line (0.6948) represents the population mean (0.388) plus 1 standard deviation (0.307). Thirteen identified compounds that displayed a fold decrease in R30E MLKL-mediated cell death ≥0.6948 (dotted line) are coloured.

To identify previously unattributed roles for protein kinases in modulating R30E MLKL constitutive death, we screened a library of 429 known kinase inhibitors (Supplementary Table S1). In contrast with conventional necroptotic signalling stimulated by TNF receptor ligation and disarming of Caspases and IAPs (Figure 2A), our screen relied only on inducing expression of R30E MLKL, which kills cells in the absence of any additional stimulus (Figure 2B). To screen for inhibitors, *MLKL^−/−^* HT29 cells were stimulated with doxycycline (100 ng/ml) to induce expression of R30E MLKL and, following overnight induction, compounds were added at 1 µM and cell death monitored by SYTOX Green uptake using IncuCyte live cell imaging (Figure 2B, Supplementary Table S1). Of 429 kinase inhibitors screened, 266 exacerbated (<0 fold decrease) R30E MLKL-mediated cell death (Figure 2C). Compounds that had a decrease in cell death equivalent to or greater than the fold decrease for GSK′872 (0.2882) minus one standard deviation (0.0855) at 24 h post-induction were considered candidate inhibitors. Fifty compounds were identified to have a one-fold decrease in R30E MLKL-mediated cell death (≥0.2027 fold; Figure 2C) and underwent further examination.

To further validate these hits, the 50 candidate compounds were screened for their capacity to inhibit R30E MLKL-mediated constitutive death when added simultaneously with doxycycline stimulation. We reasoned that the addition of these compounds at the onset of cell death, rather than 16 h post-induction as in our initial screen, would amplify any protection and allow us to more readily identify the most potent inhibitors. Of these compounds, 13 robustly inhibited R30E MLKL-mediated constitutive death (Figure 2D, Table 1). *MLKL^−/−^* HT29 cells expressing R30E MLKL and treated with AZD5363, AR-A014418, ABT-578, Tideglusib, Ipatasertib, PHA-793887, Rapamycin, Ridaforolimus, SMI-4a, LJI308, AZD1480, Idelalisib or Temsirolimus showed a one-fold decrease in cell death of ≥0.695 (mean of all samples (0.388) plus one standard deviation (0.307)) at 24 h post-stimulation (Figure 2D).

**Table 1.** Kinase inhibitors that inhibit R30E MLKL constitutive cell death

Compound	Synonyms	Primary target	Pathway	CAS ID
AZD5363	N/A	Protein kinase B (Akt)	PI3K/Akt/mTOR	1143532-39-1
AR-A014418	GSK-3β inhibitor VIII	Glycogen synthase kinase 3 (GSK-3)	PI3K/Akt/mTOR	487021-52-3
ABT-578	Zotarolimus	Mammalian target of rapamycin (mTOR)	PI3K/Akt/mTOR	221877-54-9
Tideglusib	N/A	Glycogen synthase kinase 3 (GSK-3)	PI3K/Akt/mTOR	865854-05-3
Ipatasertib	GDC-0068	Protein kinase B (Akt)	PI3K/Akt/mTOR	1001264-89-6
PHA-793887	N/A	Cyclin-dependent kinase (CDK)	Cell Cycle	718630-59-2
Rapamycin	Sirolimus	mTOR	Autophagy, DNA Damage	53123-88-9
Ridaforolimus	Deforolimus, MK-8669, AP23573	mTOR	PI3K/Akt/mTOR	572924-54-0
SMI-4a	N/A	Proto-oncogene serine/threonine protein kinase (Pim)	JAK–STAT	438190-29-5
LJI308	N/A	S6 kinase	PI3K/Akt/mTOR	1627709-94-7
AZD1480	N/A	Janus kinase 2 (JAK2)	JAK/STAT	935666-88-9
Idelalisib	CAL-101, GS-1101	Phosphoinositide 3-kinase (PI3K)	PI3K/Akt/mTOR	870281-82-6
Temsirolimus	CCI-779, NSC 683864	mTOR	Neuron signaling	162635-04-3

**Figure 3.**
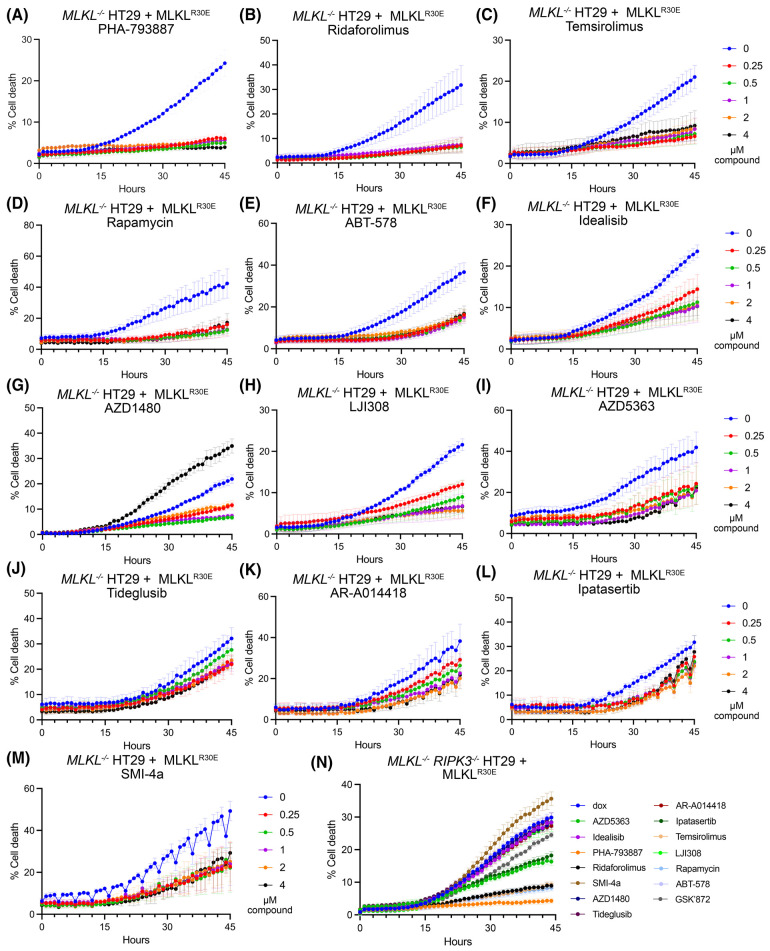
PHA-793887, Ridaforolimus, Temsirolimus, Rapamycin and ABT-578 exhibit concentration-independent inhibition of R30E MLKL death. (**A**–**M**) Evaluation of R30E MLKL constitutive death inhibition in *MLKL^−/−^*HT29 cells, following simultaneous stimulation with varying compound concentrations (0.25–4 µM) and doxycycline (100 ng/ml) to induce MLKL expression. (**N**) Evaluation of R30E MLKL constitutive death inhibition in *MLKL^−/−^* *RIPK3^−/−^* HT29 cells, following simultaneous stimulation with compound (1 µM) and doxycycline (100 ng/ml) to induce MLKL expression. Cell death was quantified over 44 h as percentage by determining the number SYTOX Green-positive cells (dead cells) relative to the number of DRAQ5-positive cells (total cell number) using IncuCyte SX5 live cell imaging. Cells were assayed in *n* = 3 (**G**, **N**), *n* = 4 (**H**, **I**, **M**), *n = 5* (**A**, **B**, **C**, **D**, **F**, **K**, **L**) or *n *= 6 (**E**, **J**) independent experiments with data plotted as mean ± SEM.

We next investigated the ability of the 13 compounds to inhibit R30E MLKL constitutive death at a range of concentrations (Figure 3A–M, Supplementary Figure S2A–M). We found that PHA-793887, Ridaforolimus, Temsirolimus, Rapamycin and ABT-578 displayed robust, concentration-independent inhibition of R30E MLKL-mediated death (Figure 3A–E, Supplementary Figure S2A–E). This protection was lost at 50 nM for PHA-793887 and 25 nM for Ridafololimus, Temsiroluimus, Rapamycin and ABT-578 (Supplementary Figure S2N). Idealisib, AZD1480 and LJI308 significantly inhibited R30E MLKL-mediated death and displayed increased inhibition at higher doses (Figure 3F–H, Supplementary Figure S2F–H). Of note, AZD1480 showed toxicity at 4 µM (Figure 3G, Supplementary Figure S2G). AZD5363, Tideglusib, AR-A014418 and Ipatasertib exhibited inhibition at higher concentrations only (Figure 3I–L, Supplementary Figure S2I–L). Treatment with SMI-4a reduced R30E MLKL constitutive cell death; however, this was not statistically significant (Figure 3M, Supplementary Figure S2M).

We also tested the ability of the 13 compounds to inhibit R30E MLKL constitutive death in *MLKL^−/−^ RIPK3^−/−^*HT29 cells at 1 µM (Figure 3N, Supplementary Figure S2O). Consistent with our findings in *MLKL^−/−^* HT29 cells, PHA-793887, Ridaforolimus, Temsirolimus, Rapamycin and ABT-578 displayed robust inhibition of R30E MLKL-mediated cell death. AZD5363 and Ipatasertib also exhibited statistically significant inhibition of constitutive cell death, whilst Idealisib, AZD1480, Tideglusib, AR-A014418 and LJI308 showed marginal reductions in cell death levels (Figure 3N, Supplementary Figure S2O). In contrast with observations in *MLKL^−/−^* HT29 cells, treatment with 1 µM of SMI-4a resulted in a slight, non-significant increase in R30E MLKL-mediated cell death. These findings are suggestive that a subset of the compounds may rely on mechanisms that involve RIPK3 to interfere with R30E MLKL-mediated cell death.

### Compounds do not inhibit cell death induced by upstream necroptotic stimuli

**Figure 4.**
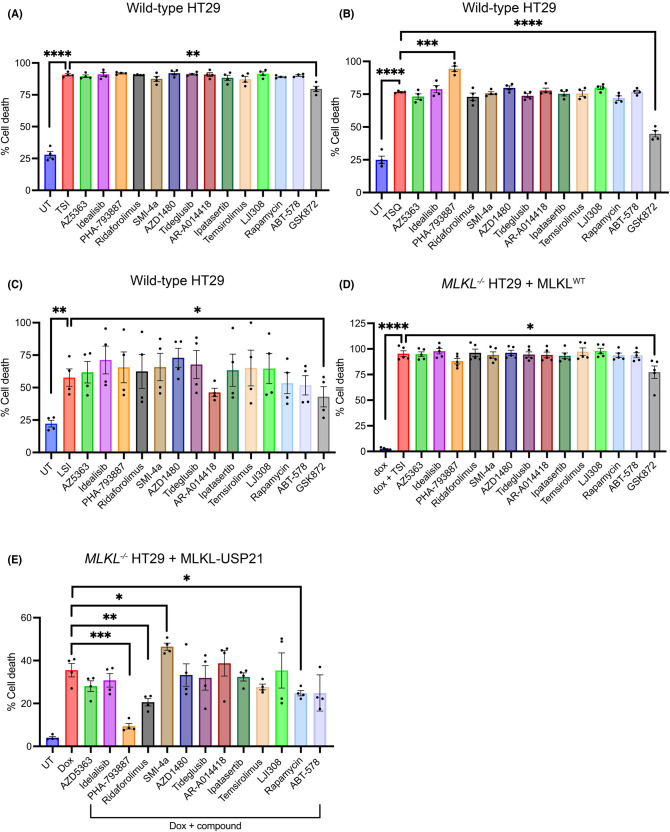
PHA-793887, Rapamycin and Ridaforolimus inhibit constitutive cell death induced by MLKL-USP21. (**A**–**C**) Evaluation of compound inhibition of necroptotic signalling in wild-type HT29 cells stimulated with necroptotic stimulus. Wild-type HT29 cells were treated with 1 µM of compound and (**A**) TSI (TNF, Smac mimetic, IDN-6556), (**B**) TSQ (TNF, Smac mimetic, QVD-OPh), or (**C**) LSI (LPS, Smac mimetic, IDN-6556). Cell death was quantified at 24 h as a percentage by determining the number SYTOX Green-positive cells (dead cells) relative to the number of DRAQ5-positive cells (total cell number) using IncuCyte SX5 live cell imaging. Cells were assayed in *n = *4 independent experiments with data plotted as mean ± SEM. (**D**) MLKL^WT^ expression was induced with doxycycline (100 ng/ml) in *MLKL^−/−^* HT29 cells treated with TSI (TNF, Smac mimetic, IDN-6556) and 1 µM of compound. (**E**) Evaluation of compound inhibition on MLKL-USP21 constitutive cell death. *MLKL^−/−^* HT29 cells were treated with doxycycline (100 ng/ml) to induce the expression of MLKL-USP21 and stimulated with 1 µM of the respective compound. Cell death was quantified at 48 h as a percentage by determining the number SYTOX Green-positive cells (dead cells) relative to the number of DRAQ5-positive cells (total cell number) using IncuCyte SX5 live cell imaging. Cells were assayed in *n = *4 (**E**) or *n *= 5 (**D**) independent experiments with data plotted as mean ± SEM. *P-*value calculated using an unpaired, two-tailed *t*-test. *P < 0.05, **P < 0.01, ***P < 0.001, ****P < 0.0001.

We next assessed whether the 13 kinase inhibitors identified to suppress R30E MLKL-mediated death in our screen could protect WT HT29 cells from necroptotic cell death induced by upstream stimuli. WT HT29 cells were stimulated with a range of necroptotic cell death inducers, of varying strengths, in the absence or presence of 1 µM of each compound. Cell death was measured by SYTOX Green uptake using IncuCyte live cell imaging at 24 h post-stimulation. We found that none of the 13 compounds showed any reductions in levels of TSI-induced cell death, whilst treatment with the RIPK3 inhibitor, GSK′872, resulted in a statistically significant reduction in when added at the time of stimulation (Figure 4A). In comparison, when compounds were tested against TSQ-induced death (TSQ: T, TNF; S, Smac mimetic Compound A; Q, QVD-OPh), known to be a weaker inducer of necroptosis than TSI [63,64], we observed that treatment with 1 µM of PHA-793887 significantly increased the levels of cell death at 24 h (Figure 4B). The remaining 12 compounds did not alter the levels of TSQ-induced death, whilst again treatment with GSK′872 resulted in a significant ∼30% reduction in necroptotic cell death (Figure 4B). Necroptotic stimulation via TLR3, which senses extracellular lipopolysaccharide (LPS), was also used to instigate cell death. WT HT29 cells were stimulated with LPS, inhibitors of cIAPs (Smac mimetic, Compound A) and Caspase-8 (IDN-6556) (LSI). Again, cells treated with LSI and 1 µM of compound exhibited similar levels of necroptotic cell death (Figure 4C). We also tested the inhibition of TSI-induced necroptotic signalling in *MLKL^−/−^* HT29 cells reconstituted with WT MLKL. Consistent with our observations in WT HT29 cells, none of the 13 compounds showed reductions in TSI-induced cell death (Figure 4D). Importantly, these findings confirm that none of the 13 compounds suppressed R30E MLKL-mediated death by interfering with doxycycline induction of MLKL exogene expression. Together, the lack of inhibition of necroptotic signalling by the 13 compounds — even upon stimulation by a range of death stimuli of different necroptotic potency — indicates these compounds do not act directly on the signalling checkpoints in the conventional necroptotic cascade.

### CDK and mTOR inhibition reduces MLKL-USP21 constitutive cell death

Based on the above results, we hypothesised that these 13 kinase inhibitors might operate on a mechanism downstream of MLKL activation that is specific to constitutively active MLKL protein. To test this idea, we examined the capacity of each kinase inhibitor to reduce the levels of necroptotic cell death executed by MLKL-USP21, known to render human MLKL constitutively active [65]. MLKL-USP21 is a fusion protein in which the catalytic domain of deubiquitylating enzyme USP21 is fused to the C-terminal end of human MLKL. This artificial fusion protein was designed to constitutively deubiquitinate MLKL and intriguingly, like R30E MLKL, MLKL-USP21 can induce necroptotic cell death independent of necroptotic stimulation.

*MLKL^−/−^* HT29 cells stably transduced with an exogene encoding MLKL-USP21 were treated with doxycycline to induce expression of the MLKL-USP21 fusion protein and treated with 1 µM of each kinase inhibitor. Cell death was measured by SYTOX Green uptake at 48 h post-stimulation using IncuCyte live cell imaging. We observed that the cyclin-dependent kinase (CDK) inhibitor, PHA-793887, robustly inhibited levels of MLKL-USP21-induced cell death (Figure 4E), consistent with the inhibition of cell death that occurred in cells expressing R30E MLKL (Figure 3A). To a lesser extent, mTOR inhibitors Ridaforolimus and Rapamycin both significantly reduced levels of MLKL-USP21-induced cell death (Figure 4E). Interestingly, SMI-4a treatment led to a significant increase in the level of cell death (Figure 4E). Consistent with this finding, SMI-4a similarly elevated cell death in *MLKL^−/−^ RIPK3^−/−^* HT29 cells expressing R30E MLKL (Figure 3N), although the underlying mechanism is currently unknown. The other nine compounds showed no statistically significant differences in the levels of cell death at 48 h (Figure 4E). Overall, our data indicate that the majority of kinase inhibitors identified herein suppress R30E MLKL-mediated cell death by targeting protein modulators of pathways distinct to those that confer constitutive activity on MLKL by removal of ubiquitylation sites.

### Compounds inhibit R30E MLKL oligomer membrane association

**Figure 5.**
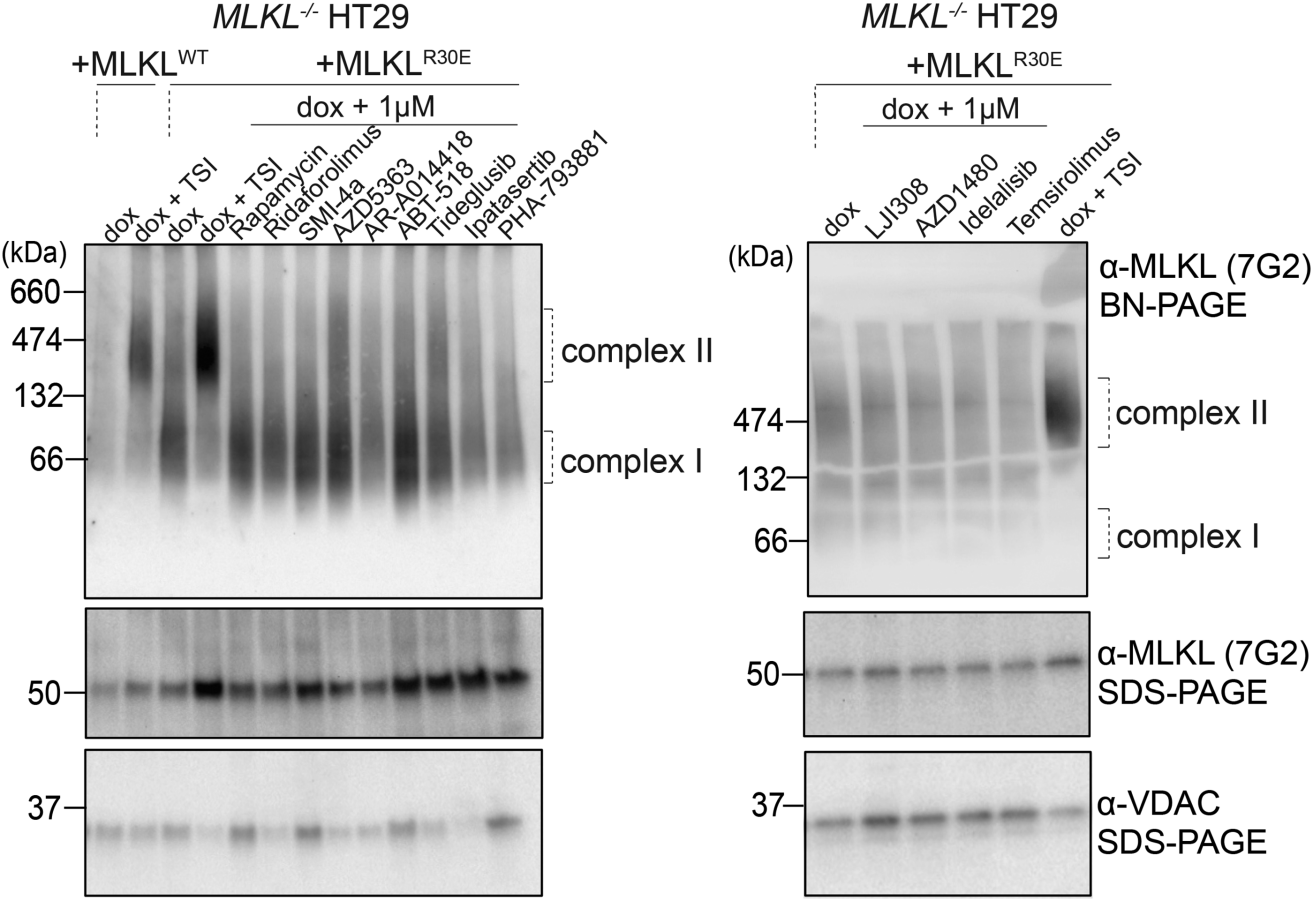
Compounds inhibit the association of R30E MLKL oligomers with the membrane. *MLKL^−/−^* HT29 cells stably transduced with *MLKL^WT^*or *MLKL^R30E^* were treated with doxycycline (Dox; 100 ng/ml) only or doxycycline plus necroptotic stimulus (Dox, TSI) in the presence or absence of kinase inhibitors (1 µM). Membrane fractions were resolved by BN-PAGE and fractionation was verified by probing for VDAC via SDS–PAGE. Images are representative of *n* = 2 independent experiments.

**Figure 6.**
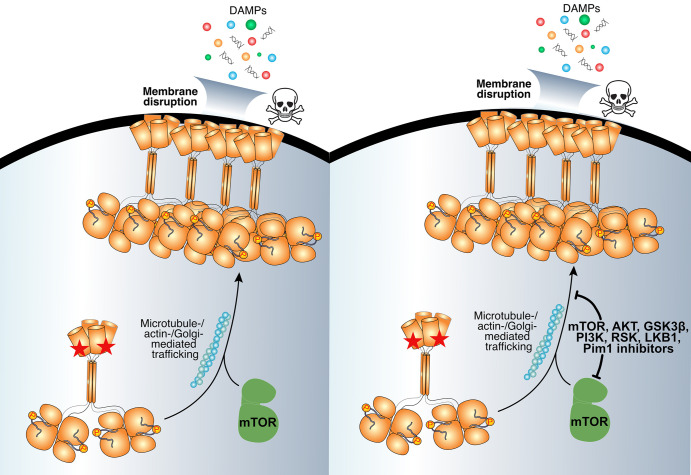
Inhibiting the mTOR pathway blocks R30E MLKL membrane translocation and cell death. Our data argue for a role for mTOR signalling in promoting R30 MLKL-mediated cell death. Inhibition of mTOR kinase activity or upstream activators of mTOR block R30E MLKL membrane translocation and killing. The skull and crossbones image (Mycomorphbox_Deadly.png; by Sven Manguard) is used via a Creative Commons Attribution-Share Alike 4.0 license.

The R30E MLKL mutation disrupts salt bridges between the 4HB domain (R30) and the brace region (E136 and D140) [59]. As a result, human R30E MLKL oligomerises into higher-order structures that associate with biological membranes, independent of RIPK3-mediated phosphorylation. We examined the effects of our compounds on the association of R30E MLKL with the membrane to determine if inhibition is mediated by disrupting the translocation of high molecular mass oligomers to the plasma membrane. *MLKL^−/−^* HT29 cells expressing MLKL (WT or R30E) were treated with compound (1 µM) or TSI stimulation and fractionated into crude membrane and cytoplasmic fractions. MLKL assembly into high molecular mass complexes was examined by Blue-Native PAGE (BN-PAGE) (Figure 5). TSI stimulation in *MLKL^−/−^* HT29 cells expressing WT or R30E MLKL resulted in high molecular mass MLKL complexes, which were present in the membrane fractions. In the absence of inhibitors, R30E MLKL expressed in *MLKL^−/−^* HT29 cells assembled into membrane-associated oligomers (Figure 5), consistent with our earlier findings [59]. Eleven of the 13 compounds identified herein blocked the translocation of high molecular mass oligomers to biological membrane, with the exceptions being AZD5363 and Tideglusib (Figure 5). These findings suggest that the primary mode of action of the inhibitors identified in our screen is to impede the association of MLKL oligomers with the plasma membrane to block R30E MLKL-mediated cell death (Figure 6).

## Discussion

Regardless of the stimulus — whether death receptor or pathogen sensor — necroptosis signalling coalesces on the core terminal effectors, RIPK1, RIPK3 and MLKL [30]. As our knowledge of the pathway has grown, there has been a greater appreciation of how signalling flux can be tuned through post-translational modification, such as by phosphorylation, ubiquitylation and acylation [45,66-71]. However, identifying protein modulators of the pathway has proven challenging. Here, to address this knowledge gap, we capitalised on the recent discovery of an activated mutant of human MLKL, R30E, which when expressed in a cell line commonly used for studies of necroptosis signalling, HT29 cells, induces cell death in the absence of any additional stimulus [59].

We screened a library of 429 kinase inhibitors for their capacity to suppress R30E MLKL-mediated cell death, and identified 13 inhibitors of diverse chemotype and reported targets. The primary mechanism by which these inhibitors prevented cell death was by blocking the translocation of R30E MLKL to the plasma membrane, which is known to precede MLKL-mediated membrane permeabilisation and is, therefore, an essential checkpoint in necroptotic signalling. Few of these 13 inhibitors have been fully characterised for specificity against a near-complete kinome panel. However, from reported targets and off-targets of these inhibitors, we deduced that mTOR signalling is a common thread in their activities, and the likely basis for suppression of R30E MLKL-mediated cell death. Intriguingly, not all mTOR inhibitors in our panel, including INK128 and MLN0128, blocked R30E MLKL-mediated cell death, suggesting additional factors, such as potency, cell permeability, mode of inhibition or off-target effects, may contribute to blocking death. Whether GSK′872 exerts its effects by targeting mTOR is currently unclear; the published profile of GSK′872 kinase specificity does not include mTOR [62]. Thus, we cannot rule out the possibility that GSK′872 inhibition of R30E MLKL-mediated cell death could also occur via off-target inhibition of mTOR.

Of the 13, four are known mTOR inhibitors: Rapamycin, Ridaforolimus, Temsirolimus and ABT-578 [72-75]. The other 9 inhibitors target kinase activators of mTOR: AKT (AZD5363, Ipatasertib [76,77]), p90 ribosomal S6 kinase (RSK; LJI308, PHA-793887 [78] and lincs.hms.harvard.edu/kinome), GSK3β (Tideglusib, AR-A014418, PHA-793887 [79,80] and lincs.hms.harvard.edu/kinome), PI3K p110 isoforms (Idelalisib [81]) and PIM1 (SMI-4a [82]). While the JAK2 inhibitor AZD1480 has not been subjected to a full kinome selectivity panel, initial screening showed off-target inhibition of LKB1, the master regulator of AMPK family kinases, at 1 μM [83]. Accordingly, LKB1 inhibition would attenuate downstream AMPK activation, which would in turn suppress mTOR signalling. Of the 13 identified compounds, those that directly targeted mTOR and the kinase activator AKT retained inhibition of R30E MLKL-mediated cell death in the absence of RIPK3. This observation suggests that indirect targeting of the mTOR signalling machinery may partially operate through a RIPK3-mediated signalling axis. However, it is important to note that while all 13 inhibitors exert effects on the mTOR signalling machinery, we cannot rule out activities on other kinases, including those for which the compounds were developed, such as PHA-793887 towards CDKs [84]. Nonetheless, the commonality of the on- and off-target effects towards mTOR pathway components provides strong evidence for a mode of action via mTOR attenuation.

The precise role of mTOR signalling in necroptotic cell death remains unclear and is an area of intense interest. To date, mTOR signalling has been primarily implicated at the level of RIPK1 or RIPK3 in necroptosis [85–88], where mTOR was reported to promote RIPK1:RIPK3 assembly into necrosomes [85,89]. To the best of our knowledge, mTOR has not been implicated in modulating necroptosis via direct MLKL regulation. Whether inhibition of mTOR signalling or an off-pathway function of the pleiotropic mTOR activator, FKBP12, underlies the blockade of necroptosis has been debated. It has been proposed that rather than mTOR signalling *per se*, blocking the non-mTOR functions of FKBP12 can diminish necroptotic signalling [89]. FKBP12 is the target of rapamycin and its analogues, such as Ridaforolimus and Temsirolimus, all of which blocked R30E MLKL-mediated death in our study. However, inhibitors that target other regulators of mTOR showed similar protection against cell death in our study, arguing for a broader function of mTOR signalling in R30E MLKL-mediated death than solely targeting FKBP12. Additionally, R30E MLKL-mediated death was also observed in cells lacking RIPK3, indicating mTOR signalling is required at the level of MLKL membrane translocation and does not exert its effects through upstream pathway effectors. Equally, R30E MLKL-mediated death did not require necroptotic stimuli, which rules out mTOR inhibition impacting Caspase-8 inactivation via the mTOR regulatory kinase, p90 RSK, which was reported to promote necroptosis in mouse cells [90]. One possibility is that inhibition of mTOR signalling compromises MLKL trafficking to the plasma membrane by impacting the Golgi and actin machinery, or potentially by dysregulating autophagic flux. Interplay between necroptosis and autophagy has been previously proposed [91], such that mTOR-mediated phosphorylation and inhibition of ULK1 kinase to block autophagy [92-94] would enable RIPK3 accumulation and poise cells to undergo necroptosis [87].

Strikingly, in contrast with previous studies [88,89], our data do not support an obligate role for mTOR signalling in promoting necroptotic cell death, even when death is triggered with different stimuli of a range of potencies. In our hands, only cell death mediated by the activated MLKL mutant, R30E, but not WT MLKL, relied on mTOR signalling. These findings argue for an auxiliary role, rather than an essential function, of mTOR in directing necroptosis. By definition, necroptosis is a pathway requiring RIPK3-mediated phosphorylation and activation of MLKL, which in turn serves as the terminal cellular executioner. However, we and others have previously noted that many animals, including many bird and rabbit species, lack a RIPK3 orthologue [6,95,96], which raises the possibility that other kinases might have been co-opted in evolution to activate MLKL for host defence or other unknown functions. The identities of such kinases remain a matter of outstanding interest, including whether mTOR-related kinases contribute to MLKL activation in species other than humans.

## Methods

### Reagents and antibodies

Primary antibodies used in this study were: rat anti-MLKL (clone 3H1 [26], produced in-house; 1:1000 dilution; available as MABC604, EMD Millipore, Billerica, MA, U.S.A.), rat anti-MLKL (clone 7G2 [41], produced in-house; 1:1000 dilution; available as MABC1636, EMD Millipore, Billerica, MA, U.S.A.) rabbit anti-human MLKL phospho-S358 (AB187091, Abcam; 1:3000), rat anti-human RIPK3 (clone 1H2 [6], produced in-house; 1:1000 dilution; available as MABC1640, EMD Millipore, Billerica, MA, U.S.A.), rabbit anti-human phospho-S227 (D6W2T, CST, 1:1000), mouse anti-actin (A-1978, Sigma–Aldrich, St Louis, MO, U.S.A.; 1:5000), rabbit anti-GAPDH (#2118, Cell Signalling; 1:2000) and rabbit anti-VDAC (AB10527, Millipore; 1:10 000). Recombinant hTNF-Fc, was produced in-house [97], and the Smac mimetic, Compound A, and pan-caspase inhibitor, IDN-6556/emricasan, were supplied by Tetralogic Pharmaceuticals. QVD-OPh was purchased from MP Biomedicals (#SKU q03OPH10901), LPS was purchased from Sigma (#L2630) and GSK′872 from SynKinase (#SYN-5481).

### Generation of cell lines

DNA sequences encoding human MLKL were synthesised by ATUM (CA, U.S.A.) and subcloned into the pFTRE3G PGK puro vector [26], as previously reported [36,40,58,59,65]. Midiprep DNA was co-transfected into HEK293T cells (ATCC) with pVSVg and pCMV δR8.2 helper plasmids to generate lentiviral particles using Effectene (Qiagen). *MLKL^−/−^* or *MLKL^−/−^ RIPK3^−/−^* HT29 cells derived from HT29 cells originally sourced from ATCC were stably transduced with the resulting lentivirus. Successful transductants were selected using puromycin (1.25–2.5 µg/ml; StemCell Technologies).

### Generation of *MLKL^−/−^ RIPK3^−/−^* HT29 cells

A full protocol for generating reconstitutable CRISPR/Cas9 knockout cell lines using integrase-deficient lentivirus has been published previously [98]. In brief, to generate the *MLKL^−/−^ RIPK3^−/−^* HT29 cell lines, a guide sequence specifically targeting the second exon of RIPK3 (GAATTCGTGCTGCGCCTAGA) [40,99] was introduced into the FgH1tUTG lentiviral expression vector. This vector was then used to produce lentivirus (as above) to transduce previously described *MLKL^−/−^* HT29 cells [58]. Guide expression was induced using 1 µg/µl doxycycline, and cell lines were sorted based on high GFP expression to ensure uptake of the lentivirus 2 days later. After a further week, cells which had failed to integrate the lentiviral vector were selected and single cell sorted based on the absence of GFP expression. The absence of RIPK3 expression was confirmed by western blotting and Illumina next-generation sequencing.

### Cell culture

Human colorectal adenocarcinoma HT29 (parental, *MLKL^−/−^*and *MLKL^−/−^ RIPK3^−/−^*) cells were cultured in high glucose DMEM (Gibco) media supplemented with 8% v/v foetal calf serum (FCS; Sigma). Puromycin (2.5 µg/ml; StemCell Technologies) was added to maintain the selection of cell lines stably transduced with inducible MLKL constructs. Routine PCR testing confirmed cell lines to be mycoplasma-negative.

### 429 Kinase inhibitor compound screen

*MLKL^−/−^* HT29 cells were seeded into 96-well flat bottom plates at 2 × 10^4 ^cells/well and left to settle for 6–8 h prior to treatment with doxycycline (100 ng/ml) overnight (∼16 h) to induce expression of R30E MLKL constructs. Cells were then treated with 1 µM of test compound or 10 µM of GSK′872 and fresh doxycycline (100 ng/ml) in DMEM supplemented with 2% FCS, SYTOX Green (Invitrogen S7020; 1:10 000) and DRAQ5 (ThermoFisher Scientific #62251; 1:5000). Cells were moved into the IncuCyte SX5 System (Essen Bioscience; v2022B) and images captured every hour. Cell death percentage values were quantified by number of SYTOX Green-positive (dead) cells out of DRAQ5-positive (total) cell numbers. Compounds were assayed in technical duplicates.

### Incucyte cell death assays

For rescreening of 50 candidate compounds, *MLKL^−/−^*HT29 cells stably transduced with R30E MLKL were seeded into 96-well flat bottom plates at 2 × 10^4 ^cells/well and left to settle for 6–8 h prior to treatment with doxycycline (100 ng/ml) and 1 µM compound. The next morning, media was removed and replaced with fresh DMEM supplemented with 2% FCS, SYTOX Green (Invitrogen S7020; 1:10 000), DRAQ5 (ThermoFisher Scientific #62251; 1:5000), doxycycline (100 ng/ml) and 1 µM of compound. Cells were moved into the IncuCyte SX5 System (Essen Bioscience; v2022B) and cell death percentage values were quantified by number of SYTOX Green-positive (dead) cells out of DRAQ5-positive (total) cell numbers. Fold change was quantified as ratio of R30E MLKL-mediated cell death with no compound to compound.

*MLKL^−/−^ RIPK3^−/−^* HT29 cells were seeded into 48-well plate at 4.5 × 10^4 ^cells/well and left to settle for 6–8 h prior to treatment with doxycycline (100 ng/ml) overnight to induce expression of WT or R30E MLKL constructs. Following overnight doxycycline induction, *MLKL^−/−^ RIPK3^−/−^* HT29 cells were stimulated with the outlined combination treatments of doxycycline (100 ng/ml), TNF (100 ng/ml), Smac mimetic Compound A (500 nM), pan-caspase inhibitor IDN-6556 (5 µM) and GSK′872 in DMEM supplemented with 2% FCS, SYTOX Green (Invitrogen S7020; 1:10 000) and DRAQ5 (ThermoFisher Scientific #62251; 1:5000).

*MLKL^−/−^* or *MLKL^−/−^ RIPK3^−/−^* HT29 stably transduced with WT or R30E MLKL were seeded into 96-well plates at 2.0 × 10^4 ^cells/well and *MLKL^−/−^* HT29 stably transduced MLKL-USP21 were seeded into 96-well plates at 1.5 × 10^4^ cells/well. All cells were left to settle for 6–8 h. For compound titration (including GSK′872) and MLKL-USP21 experiments, cells were stimulated with doxycycline (100 ng/ml) and compounds (0.025–10 µM) in DMEM supplemented with 2% FCS, SYTOX Green (Invitrogen S7020; 1:10 000) and DRAQ5 (ThermoFisher Scientific #62251) and plates were moved immediately into the IncuCyte SX5 System (Essen Bioscience; v2022B). Images were taken every hour and cell death percentage values were quantified by number of SYTOX Green-positive (dead) cells out of DRAQ5-positive (total) cell numbers.

For assessment of response to necroptotic death stimuli, WT HT29 cells were seeded into 96-well plates at 2.0 × 10^4^ cells/well and left to settle for 6–8 h. Cells were then stimulated with combination treatments TSI, TSQ or LSI (TNF (100 ng/ml), Smac mimetic Compound A (500 nM), pan-caspase inhibitor IDN-6556 (5 µM), QVD-OPh (10 µM), LPS (20 ng/ml)) in the presence or absence of compound (1 µM; 10 µM GSK′872) in DMEM supplemented with 2% FCS, SYTOX Green (Invitrogen S7020; 1:10 000) and DRAQ5 (ThermoFisher Scientific #62251). Plates were moved into the IncuCyte SX5 System (Essen Bioscience; v2022B) and cell death percentage values were quantified by number of SYTOX Green-positive (dead) cells out of DRAQ5-positive (total) cell numbers.

### Western blot

*MLKL^−/−^ RIPK3^−/−^* HT29 cells were seeded into 48-well plates at 6 × 10^4 ^cells/well and left to settle for 6–8 h. Cells were stimulated with doxycycline (100 ng/ml) overnight to induce human MLKL expression. Cells were harvested in 2× SDS Laemmli reducing lysis buffer, boiled at 100°C for 10–15 min, and then resolved by 4–15% Tris-Glycine gel (Bio-Rad). Proteins were transferred to the PVDF membrane and probed with antibodies as indicated.

### BN-PAGE

*MLKL^−/−^* HT29 cells were seeded at 0.6 × 10^6 ^cells/well in a 6-well plate and left to settle overnight. The next day, cells were induced with doxycycline (100 ng/ml) and compound (1 µM) overnight. Where applicable, cells were treated with necroptotic stimulants — TNF (100 ng/ml), Smac mimetic Compound A (500 nM), and Pan-caspase inhibitor IDN-6556 (5 µM) — for 4.5 h. Cells were harvested and permeabilised in MELB buffer (20 mM HEPES pH 7.5, 100 mM sucrose, 100 mM KCl, 2 µM *N*-ethyl maleimide, 2.5 mM MgCl_2_, 0.25% (v/v) digitonin, and protease and phosphatase inhibitors). Cells were fractionated into cytoplasmic and crude membrane fractions by centrifugation (5 min, 11 000×***g***) and solubilised in 1% digitonin. Samples were resolved on a 4–16% NativePAGE (Invitrogen) gel and proteins were transferred to the PVDF membrane. Membranes were destained in 50% v/v methanol and 25% v/v acetic acid and then denatured (6 M guanidine hydrochloride, 10 mM Tris PH 6.8, 5 mM β-mercaptoethanol). Following blocking in 5% w/v skim milk, membranes were probed with indicated antibodies.

## Data Availability

All data and reagents are available from the authors upon request. Uncropped western blots are included as Supplementary Data.

## Open Access

Open access for this article was enabled by the participation of University of Melbourne in an all-inclusive *Read & Publish* agreement with Portland Press and the Biochemical Society under a transformative agreement with CAUL.

## Abbreviations

CDK: cyclin-dependent kinase
cIAP: cellular inhibitor of apoptosis proteins
DAMPs: damage-associated molecular patterns
LPS: lipopolysaccharide
MLKL: mixed lineage kinase domain-like
RIPK: receptor-interacting serine/threonine protein kinase
TLR: Toll-like receptors
WT: wild-type

## References

[1] Nakano, H., Murai, S. and Moriwaki, K. (2022) Regulation of the release of damage-associated molecular patterns from necroptotic cells. Biochem. J. 479, 677–685 10.1042/BCJ20210604 35293986

[2] Fletcher-Etherington, A., Nobre, L., Nightingale, K., Antrobus, R., Nichols, J., Davison, A.J. et al. (2020) Human cytomegalovirus protein pUL36: a dual cell death pathway inhibitor. Proc. Natl Acad. Sci. U.S.A. 117, 18771–18779 10.1073/pnas.2001887117 32690704 PMC7414183

[3] Kitur, K., Wachtel, S., Brown, A., Wickersham, M., Paulino, F., Penaloza, H.F. et al. (2016) Necroptosis promotes *Staphylococcus aureus* clearance by inhibiting excessive inflammatory signaling. Cell Rep. 16, 2219–2230 10.1016/j.celrep.2016.07.039 27524612 PMC5001919

[4] Liu, Z., Nailwal, H., Rector, J., Rahman, M.M., Sam, R., McFadden, G. et al. (2021) A class of viral inducer of degradation of the necroptosis adaptor RIPK3 regulates virus-induced inflammation. Immunity 54, 247–258.e7. 10.1016/j.immuni.2020.11.020 33444549 PMC7878414

[5] Pearson, J.S., Giogha, C., Muhlen, S., Nachbur, U., Pham, C.L., Zhang, Y. et al. (2017) Espl is a bacterial cysteine protease effector that cleaves RHIM proteins to block necroptosis and inflammation. Nat. Microbiol. 2, 16258 10.1038/nmicrobiol.2016.258 28085133 PMC7613272

[6] Petrie, E.J., Sandow, J.J., Lehmann, W.I.L., Liang, L.Y., Coursier, D., Young, S.N. et al. (2019) Viral MLKL homologs subvert necroptotic cell death by sequestering cellular RIPK3. Cell Rep. 28, 3309–3319.e5. 10.1016/j.celrep.2019.08.055 31553902

[7] Chiou, S., Al-Ani, A.H., Pan, Y., Patel, K.M., Kong, I.Y., Whitehead, L.W. et al. (2024) An immunohistochemical atlas of necroptotic pathway expression. 16, 1717–1749. 10.1038/s44321-024-00074-6 PMC11250867 38750308

[8] Gong, Y.N., Guy, C., Olauson, H., Becker, J.U., Yang, M., Fitzgerald, P. et al. (2017) ESCRT-III acts downstream of MLKL to regulate necroptotic cell death and its consequences. Cell 169, 286–300.e16. 10.1016/j.cell.2017.03.020 28388412 PMC5443414

[9] Kolbrink, B., von Samson-Himmelstjerna, F.A., Murphy, J.M., Krautwald, S. (2023) Role of necroptosis in kidney health and disease. Nat. Rev. Nephrol. 19, 300–314 10.1038/s41581-022-00658-w 36596919

[10] Pierdomenico, M., Negroni, A., Stronati, L., Vitali, R., Prete, E., Bertin, J. et al. (2014) Necroptosis is active in children with inflammatory bowel disease and contributes to heighten intestinal inflammation. Am. J. Gastroenterol. 109, 279–287 10.1038/ajg.2013.403 24322838

[11] Wang, H., Sun, L., Su, L., Rizo, J., Liu, L., Wang, L.F. et al. (2014) Mixed lineage kinase domain-like protein MLKL causes necrotic membrane disruption upon phosphorylation by RIP3. Mol. Cell 54, 133–146 10.1016/j.molcel.2014.03.003 24703947

[12] Lu, Z., Van Eeckhoutte, H.P., Liu, G., Nair, P.M., Jones, B., Gillis, C.M. et al. (2021) Necroptosis signaling promotes inflammation, airway remodeling, and emphysema in chronic obstructive pulmonary disease. Am. J. Respir. Crit. Care Med. 204, 667–681 10.1164/rccm.202009-3442OC 34133911

[13] Faergeman, S.L., Evans, H., Attfield, K.E., Desel, C., Kuttikkatte, S.B., Sommerlund, M. et al. (2020) A novel neurodegenerative spectrum disorder in patients with MLKL deficiency. Cell Death Dis. 11, 303 10.1038/s41419-020-2494-0 32358523 PMC7195448

[14] Hildebrand, J.M., Kauppi, M., Majewski, I.J., Liu, Z., Cox, A.J., Miyake, S. et al. (2020) A missense mutation in the MLKL brace region promotes lethal neonatal inflammation and hematopoietic dysfunction. Nat. Commun. 11, 3150 10.1038/s41467-020-16819-z 32561755 PMC7305203

[15] Garnish, S.E. and Hildebrand, J.M. (2022) Rare catastrophes and evolutionary legacies: human germline gene variants in MLKL and the necroptosis signalling pathway. Biochem. Soc. Trans. 50, 529–539 10.1042/BST20210517 35166320 PMC9022980

[16] Hildebrand, J.M., Lo, B., Tomei, S., Mattei, V., Young, S.N., Fitzgibbon, C. et al. (2021) A family harboring an MLKL loss of function variant implicates impaired necroptosis in diabetes. Cell Death Dis. 12, 345 10.1038/s41419-021-03636-5 33795639 PMC8016849

[17] Garnish, S.E., Martin, K.R., Kauppi, M., Jackson, V.E., Ambrose, R., Eng, V.V. et al. (2023) A common human MLKL polymorphism confers resistance to negative regulation by phosphorylation. Nat. Commun. 14, 6046 10.1038/s41467-023-41724-6 37770424 PMC10539340

[18] Lawlor, K.E., Murphy, J.M. and Vince, J.E. (2024) Gasdermin and MLKL necrotic cell death effectors: signaling and diseases. Immunity 57, 429–445 10.1016/j.immuni.2024.02.011 38479360

[19] Newton, K., Dugger, D.L., Maltzman, A., Greve, J.M., Hedehus, M., Martin-McNulty, B. et al. (2016) RIPK3 deficiency or catalytically inactive RIPK1 provides greater benefit than MLKL deficiency in mouse models of inflammation and tissue injury. Cell Death Differ. 23, 1565–1576 10.1038/cdd.2016.46 27177019 PMC5072432

[20] Rickard, J.A., Anderton, H., Etemadi, N., Nachbur, U., Darding, M., Peltzer, N. et al. (2014) TNFR1-dependent cell death drives inflammation in Sharpin-deficient mice. Elife 3, e03464 10.7554/eLife.03464 25443632 PMC4270099

[21] Rickard, J.A., O'Donnell, J.A., Evans, J.M., Lalaoui, N., Poh, A.R., Rogers, T. et al. (2014) RIPK1 regulates RIPK3-MLKL-driven systemic inflammation and emergency hematopoiesis. Cell 157, 1175–1188 10.1016/j.cell.2014.04.019 24813849

[22] Vucur, M., Ghallab, A., Schneider, A.T., Adili, A., Cheng, M., Castoldi, M. et al. (2023) Sublethal necroptosis signaling promotes inflammation and liver cancer. Immunity 56, 1578–1595.e8. 10.1016/j.immuni.2023.05.017 37329888

[23] Wu, J., Huang, Z., Ren, J., Zhang, Z., He, P., Li, Y. et al. (2013) Mlkl knockout mice demonstrate the indispensable role of Mlkl in necroptosis. Cell Res. 23, 994–1006 10.1038/cr.2013.91 23835476 PMC3731568

[24] Garcia, L.R., Tenev, T., Newman, R., Haich, R.O., Liccardi, G., John, S.W. et al. (2021) Ubiquitylation of MLKL at lysine 219 positively regulates necroptosis-induced tissue injury and pathogen clearance. Nat. Commun. 12, 3364 10.1038/s41467-021-23474-5 34099649 PMC8184782

[25] Tovey Crutchfield, E.C., Garnish, S.E. and Hildebrand, J.M. (2021) The role of the key effector of necroptotic cell death, MLKL, in mouse models of disease. Biomolecules 11, 803 10.3390/biom11060803 34071602 PMC8227991

[26] Murphy, J.M., Czabotar, P.E., Hildebrand, J.M., Lucet, I.S., Zhang, J.G., Alvarez-Diaz, S. et al. (2013) The pseudokinase MLKL mediates necroptosis via a molecular switch mechanism. Immunity 39, 443–453 10.1016/j.immuni.2013.06.018 24012422

[27] Newton, K., Sun, X. and Dixit, V.M. (2004) Kinase RIP3 is dispensable for normal NF-kappa Bs, signaling by the B-cell and T-cell receptors, tumor necrosis factor receptor 1, and Toll-like receptors 2 and 4. Mol. Cell Biol. 24, 1464–1469 10.1128/MCB.24.4.1464-1469.2004 14749364 PMC344190

[28] Crutchfield, T., Garnish, E.C., Day, S.E., Anderton, J., Chiou, H., Hempel, S. (2023) MLKL deficiency protects against low-grade, sterile inflammation in aged mice. Cell Death Differ. 30, 1059–1071 10.1038/s41418-023-01121-4 36755069 PMC10070424

[29] Horne, C.R., Samson, A.L. and Murphy, J.M. (2023) The web of death: the expanding complexity of necroptotic signaling. Trends Cell Biol. 33, 162–174 10.1016/j.tcb.2022.05.008 35750616

[30] Samson, A.L., Garnish, S.E., Hildebrand, J.M. and Murphy, J.M. (2021) Location, location, location: a compartmentalized view of TNF-induced necroptotic signaling. Sci. Signal. 14, eabc6178 10.1126/scisignal.abc6178 33531383

[31] Degterev, A., Huang, Z., Boyce, M., Li, Y., Jagtap, P., Mizushima, N. et al. (2005) Chemical inhibitor of nonapoptotic cell death with therapeutic potential for ischemic brain injury. Nat. Chem. Biol. 1, 112–119 10.1038/nchembio711 16408008

[32] Holler, N., Zaru, R., Micheau, O., Thome, M., Attinger, A., Valitutti, S. et al. (2000) Fas triggers an alternative, caspase-8-independent cell death pathway using the kinase RIP as effector molecule. Nat. Immunol. 1, 489–495 10.1038/82732 11101870

[33] Kaiser, W.J., Sridharan, H., Huang, C., Mandal, P., Upton, J.W., Gough, P.J. et al. (2013) Toll-like receptor 3-mediated necrosis via TRIF. RIP **3**, and MLKL. J. Biol. Chem. 288, 31268–31279. 10.1074/jbc.M113.462341 24019532 PMC3829437

[34] Lin, J., Kumari, S., Kim, C., Van, T.M., Wachsmuth, L., Polykratis, A. et al. (2016) RIPK1 counteracts ZBP1-mediated necroptosis to inhibit inflammation. Nature 540, 124–128 10.1038/nature20558 27819681 PMC5755685

[35] Newton, K., Wickliffe, K.E., Maltzman, A., Dugger, D.L., Strasser, A., Pham, V.C. et al. (2016) RIPK1 inhibits ZBP1-driven necroptosis during development. Nature 540, 129–133 10.1038/nature20559 27819682

[36] Meng, Y., Davies, K.A., Fitzgibbon, C., Young, S.N., Garnish, S.E., Horne, C.R. et al. (2021) Human RIPK3 maintains MLKL in an inactive conformation prior to cell death by necroptosis. Nat. Commun. 12, 6783 10.1038/s41467-021-27032-x 34811356 PMC8608796

[37] Sun, L., Wang, H., Wang, Z., He, S., Chen, S., Liao, D. et al. (2012) Mixed lineage kinase domain-like protein mediates necrosis signaling downstream of RIP3 kinase. Cell 148, 213–227 10.1016/j.cell.2011.11.031 22265413

[38] Li, J., McQuade, T., Siemer, A.B., Napetschnig, J., Moriwaki, K., Hsiao, Y.S. et al. (2012) The RIP1/RIP3 necrosome forms a functional amyloid signaling complex required for programmed necrosis. Cell 150, 339–350 10.1016/j.cell.2012.06.019 22817896 PMC3664196

[39] Mompean, M., Li, W., Li, J., Laage, S., Siemer, A.B., Bozkurt, G. et al. (2018) The structure of the necrosome RIPK1-RIPK3 core, a human hetero-amyloid signaling complex. Cell 173, 1244–1253.e10. 10.1016/j.cell.2018.03.032 29681455 PMC6002806

[40] Garnish, S.E., Meng, Y., Koide, A., Sandow, J.J., Denbaum, E., Jacobsen, A.V. et al. (2021) Conformational interconversion of MLKL and disengagement from RIPK3 precede cell death by necroptosis. Nat. Commun. 12, 2211 10.1038/s41467-021-22400-z 33850121 PMC8044208

[41] Samson, A.L., Zhang, Y., Geoghegan, N.D., Gavin, X.J., Davies, K.A., Mlodzianoski, M.J. et al. (2020) MLKL trafficking and accumulation at the plasma membrane control the kinetics and threshold for necroptosis. Nat. Commun. 11, 3151 10.1038/s41467-020-16887-1 32561730 PMC7305196

[42] Fan, W., Guo, J., Gao, B., Zhang, W., Ling, L., Xu, T. et al. (2019) Flotillin-mediated endocytosis and ALIX-syntenin-1-mediated exocytosis protect the cell membrane from damage caused by necroptosis. Sci. Signal. 12, eaaw3423 10.1126/scisignal.aaw3423 31138766

[43] Yoon, S., Kovalenko, A., Bogdanov, K. and Wallach, D. (2017) MLKL, the protein that mediates necroptosis, also regulates endosomal trafficking and extracellular vesicle generation. Immunity 47, 51–65.e7. 10.1016/j.immuni.2017.06.001 28666573

[44] Zargarian, S., Shlomovitz, I., Erlich, Z., Hourizadeh, A., Ofir-Birin, Y., Croker, B.A. et al. (2017) Phosphatidylserine externalization, “necroptotic bodies” release, and phagocytosis during necroptosis. PLoS Biol. 15, e2002711 10.1371/journal.pbio.2002711 28650960 PMC5501695

[45] Zhu, X., Yang, N., Yang, Y., Yuan, F., Yu, D., Zhang, Y. et al. (2022) Spontaneous necroptosis and autoinflammation are blocked by an inhibitory phosphorylation on MLKL during neonatal development. Cell Res. 32, 407–410 10.1038/s41422-021-00583-w 34728815 PMC8975819

[46] Hanna-Addams, S., Liu, S., Liu, H., Chen, S. and Wang, Z. (2020) CK1alpha, CK1delta, and CK1epsilon are necrosome components which phosphorylate serine 227 of human RIPK3 to activate necroptosis. Proc. Natl Acad. Sci. U.S.A. 117, 1962–1970 10.1073/pnas.1917112117 31932442 PMC6995002

[47] Najafov, A., Mookhtiar, A.K., Luu, H.S., Ordureau, A., Pan, H., Amin, P.P. et al. (2019) TAM kinases promote necroptosis by regulating oligomerization of MLKL. Mol. Cell 75, 457–468.e4. 10.1016/j.molcel.2019.05.022 31230815

[48] Reynoso, E., Liu, H., Li, L., Yuan, A.L., Chen, S. and Wang, Z. (2017) Thioredoxin-1 actively maintains the pseudokinase MLKL in a reduced state to suppress disulfide bond-dependent MLKL polymer formation and necroptosis. J. Biol Chem. 292, 17514–17524 10.1074/jbc.M117.799353 28878015 PMC5655526

[49] Liu, W., Chen, B., Wang, Y., Meng, C., Huang, H., Huang, X.R. et al. (2018) RGMb protects against acute kidney injury by inhibiting tubular cell necroptosis via an MLKL-dependent mechanism. Proc. Natl Acad. Sci. U.S.A. 115, E1475–E1E84 10.1073/pnas.1716959115 29382757 PMC5816182

[50] Jacobsen, A.V., Lowes, K.N., Tanzer, M.C., Lucet, I.S., Hildebrand, J.M., Petrie, E.J. et al. (2016) HSP90 activity is required for MLKL oligomerisation and membrane translocation and the induction of necroptotic cell death. Cell Death Dis. 7, e2051 10.1038/cddis.2015.386 26775703 PMC4816171

[51] Bigenzahn, J.W., Fauster, A., Rebsamen, M., Kandasamy, R.K., Scorzoni, S., Vladimer, G.I. et al. (2016) An inducible retroviral expression system for tandem affinity purification mass-spectrometry-based proteomics identifies mixed lineage kinase domain-like protein (MLKL) as an heat shock protein 90 (HSP90) client. Mol. Cell. Proteomics 15, 1139–1150 10.1074/mcp.o115.055350 26933192 PMC4813694

[52] Lewis, J., Devin, A., Miller, A., Lin, Y., Rodriguez, Y., Neckers, L. et al. (2000) Disruption of hsp90 function results in degradation of the death domain kinase, receptor-interacting protein (RIP), and blockage of tumor necrosis factor-induced nuclear factor-kappaB activation. J. Biol. Chem. 275, 10519–10526 10.1074/jbc.275.14.10519 10744744

[53] Li, D., Xu, T., Cao, Y., Wang, H., Li, L., Chen, S. et al. (2015) A cytosolic heat shock protein 90 and cochaperone CDC37 complex is required for RIP3 activation during necroptosis. Proc. Natl Acad. Sci. U.S.A. 112, 5017–5022 10.1073/pnas.1505244112 25852146 PMC4413296

[54] Park, S.Y., Shim, J.H. and Cho, Y.S. (2014) Distinctive roles of receptor-interacting protein kinases 1 and 3 in caspase-independent cell death of L929. Cell Biochem. Funct. 32, 62–69 10.1002/cbf.2972 23584955

[55] Zhao, X.M., Chen, Z., Zhao, J.B., Zhang, P.P., Pu, Y.F., Jiang, S.H. et al. (2016) Hsp90 modulates the stability of MLKL and is required for TNF-induced necroptosis. Cell Death Dis. 7, e2089 10.1038/cddis.2015.390 26866270 PMC4849146

[56] Jacobsen, A.V., Pierotti, C.L., Lowes, K.N., Au, A.E., Zhang, Y., Etemadi, N. et al. (2022) The Lck inhibitor, AMG, 47a, blocks necroptosis and implicates RIPK1 in signalling downstream of MLKL. Cell Death Dis. 13, 291. 10.1038/s41419-022-04740-w 35365636 PMC8976052

[57] Pierotti, C.L., Jacobsen, A.V., Grohmann, C., Dempsey, R.K., Etemadi, N., Hildebrand, J.M. et al. (2023) The VEGFR/PDGFR tyrosine kinase inhibitor. ABT 869, blocks necroptosis by targeting RIPK1 kinase. Biochem. J. 480, 665–684. 10.1042/BCJ20230035 37115711 PMC10212518

[58] Petrie, E.J., Sandow, J.J., Jacobsen, A.V., Smith, B.J., Griffin, M.D.W., Lucet, I.S. et al. (2018) Conformational switching of the pseudokinase domain promotes human MLKL tetramerization and cell death by necroptosis. Nat. Commun. 9, 2422 10.1038/s41467-018-04714-7 29930286 PMC6013482

[59] Meng, Y., Garnish, S.E., Davies, K.A., Black, K.A., Leis, A.P., Horne, C.R. et al. (2023) Phosphorylation-dependent pseudokinase domain dimerization drives full-length MLKL oligomerization. Nat. Commun. 14, 6804 10.1038/s41467-023-42255-w 37884510 PMC10603135

[60] Landrum, M.J., Lee, J.M., Riley, G.R., Jang, W., Rubinstein, W.S., Church, D.M. et al. (2014) Clinvar: public archive of relationships among sequence variation and human phenotype. Nucleic Acids Res. 42, D980–D985 10.1093/nar/gkt1113 24234437 PMC3965032

[61] Sondka, Z., Dhir, N.B., Carvalho-Silva, D., Jupe, S., Madhumita, M. and et al., K. (2024) COSMIC: a curated database of somatic variants and clinical data for cancer. Nucleic Acids Res. 52, D1210–D12D7 10.1093/nar/gkad986 38183204 PMC10767972

[62] Mandal, P., Berger, S.B., Pillay, S., Moriwaki, K., Huang, C., Guo, H. et al. (2014) RIP3 induces apoptosis independent of pronecrotic kinase activity. Mol. Cell 56, 481–495 10.1016/j.molcel.2014.10.021 25459880 PMC4512186

[63] Brumatti, G., Ma, C., Lalaoui, N., Nguyen, N.Y., Navarro, M., Tanzer, M.C. et al. (2016) The caspase-8 inhibitor emricasan combines with the SMAC mimetic birinapant to induce necroptosis and treat acute myeloid leukemia. Sci. Transl. Med. 8, a69 10.1126/scitranslmed.aad3099 27194727

[64] Pierotti, C.L., Tanzer, M.C., Jacobsen, A.V., Hildebrand, J.M., Garnier, J.M., Sharma, P. et al. (2020) Potent inhibition of necroptosis by simultaneously targeting multiple effectors of the pathway. ACS Chem. Biol. 15, 2702–2713 10.1021/acschembio.0c00482 32902249

[65] Liu, Z., Dagley, L.F., Shield-Artin, K., Young, S.N., Bankovacki, A., Wang, X. et al. (2021) Oligomerization-driven MLKL ubiquitylation antagonizes necroptosis. EMBO J. 40, e103718 10.15252/embj.2019103718 34698396 PMC8634140

[66] Meng, Y., Sandow, J.J., Czabotar, P.E. and Murphy, J.M. (2021) The regulation of necroptosis by post-translational modifications. Cell Death Differ. 28, 861–883 10.1038/s41418-020-00722-7 33462412 PMC7937688

[67] Pradhan, A.J., Chitkara, S., Ramirez, R.X., Monje-Galvan, V., Sancak, Y. and Atilla-Gokcumen, G.E. (2024) Acylation of MLKL impacts its function in necroptosis. ACS Chem. Biol. 19, 407–418 10.1021/acschembio.3c00603 38301282

[68] Pradhan, A.J., Lu, D., Parisi, L.R., Shen, S., Berhane, I.A., Galster, S.L. et al. (2021) Protein acylation by saturated very long chain fatty acids and endocytosis are involved in necroptosis. Cell Chem. Biol. 28, 1298–1309.e7. 10.1016/j.chembiol.2021.03.012 33848465 PMC8529612

[69] Meng, Y., Horne, C.R., Samson, A.L., Dagley, L.F., Young, S.N., Sandow, J.J. et al. (2022) Human RIPK3 C-lobe phosphorylation is essential for necroptotic signaling. Cell Death Dis. 13, 565 10.1038/s41419-022-05009-y 35739084 PMC9226014

[70] Roedig, J., Kowald, L., Juretschke, T., Karlowitz, R., Ahangarian Abhari, B., Roedig, H. et al. (2021) USP22 controls necroptosis by regulating receptor-interacting protein kinase 3 ubiquitination. EMBO Rep. 22, e50163 10.15252/embr.202050163 33369872 PMC7857539

[71] Weinelt, N., Wachtershauser, K.N., Celik, G., Jeiler, B., Gollin, I., Zein, L. et al. (2024) LUBAC-mediated M1 Ub regulates necroptosis by segregating the cellular distribution of active MLKL. Cell Death Dis. 15, 77 10.1038/s41419-024-06447-6 38245534 PMC10799905

[72] Lorenz, M.C. and Heitman, J. (1995) TOR mutations confer rapamycin resistance by preventing interaction with FKBP12-rapamycin. J. Biol. Chem. 270, 27531–27537 10.1074/jbc.270.46.27531 7499212

[73] Sabers, C.J., Martin, M.M., Brunn, G.J., Williams, J.M., Dumont, F.J., Wiederrecht, G. et al. (1995) Isolation of a protein target of the FKBP12-rapamycin complex in mammalian cells. J. Biol. Chem. 270, 815–822 10.1074/jbc.270.2.815 7822316

[74] Vignot, S., Faivre, S., Aguirre, D. and Raymond, E. (2005) mTOR-targeted therapy of cancer with rapamycin derivatives. Ann. Oncol. 16, 525–537 10.1093/annonc/mdi113 15728109

[75] Wagner, R., Mollison, K.W., Liu, L., Henry, C.L., Rosenberg, T.A., Bamaung, N. et al. (2005) Rapamycin analogs with reduced systemic exposure. Bioorg. Med. Chem. Lett. 15, 5340–5343 10.1016/j.bmcl.2005.06.106 16185865

[76] Davies, B.R., Greenwood, H., Dudley, P., Crafter, C., Yu, D.H., Zhang, J. et al. (2012) Preclinical pharmacology of AZD5363, an inhibitor of AKT: pharmacodynamics, antitumor activity, and correlation of monotherapy activity with genetic background. Mol. Cancer Ther. 11, 873–887 10.1158/1535-7163.MCT-11-0824-T 22294718

[77] Blake, J.F., Xu, R., Bencsik, J.R., Xiao, D., Kallan, N.C., Schlachter, S. et al. (2012) Discovery and preclinical pharmacology of a selective ATP-competitive Akt inhibitor (GDC-0068) for the treatment of human tumors. J. Med. Chem. 55, 8110–8127 10.1021/jm301024w 22934575

[78] Aronchik, I., Appleton, B.A., Basham, S.E., Crawford, K., Del Rosario, M., Doyle, L.V. et al. (2014) Novel potent and selective inhibitors of p90 ribosomal S6 kinase reveal the heterogeneity of RSK function in MAPK-driven cancers. Mol. Cancer Res. 12, 803–812 10.1158/1541-7786.MCR-13-0595 24554780

[79] Dominguez, J.M., Fuertes, A., Orozco, L., del Monte-Millan, M., Delgado, E. and Medina, M. (2012) Evidence for irreversible inhibition of glycogen synthase kinase-3β by tideglusib. J. Biol. Chem. 287, 893–904 10.1074/jbc.M111.306472 22102280 PMC3256883

[80] Bhat, R., Xue, Y., Berg, S., Hellberg, S., Ormo, M., Nilsson, Y. et al. (2003) Structural insights and biological effects of glycogen synthase kinase 3-specific inhibitor AR-A014418. J. Biol. Chem. 278, 45937–45945 10.1074/jbc.M306268200 12928438

[81] Lannutti, B.J., Meadows, S.A., Herman, S.E., Kashishian, A., Steiner, B., Johnson, A.J. et al. (2011) CAL-101, a p110delta selective phosphatidylinositol-3-kinase inhibitor for the treatment of B-cell malignancies, inhibits PI3K signaling and cellular viability. Blood 117, 591–594 10.1182/blood-2010-03-275305 20959606 PMC3694505

[82] Xia, Z., Knaak, C., Ma, J., Beharry, Z.M., McInnes, C., Wang, W. et al. (2009) Synthesis and evaluation of novel inhibitors of Pim-1 and Pim-2 protein kinases. J. Med. Chem. 52, 74–86 10.1021/jm800937p 19072652 PMC5404933

[83] Hedvat, M., Huszar, D., Herrmann, A., Gozgit, J.M., Schroeder, A., Sheehy, A. et al. (2009) The JAK2 inhibitor AZD1480 potently blocks Stat3 signaling and oncogenesis in solid tumors. Cancer Cell 16, 487–497 10.1016/j.ccr.2009.10.015 19962667 PMC2812011

[84] Brasca, M.G., Albanese, C., Alzani, R., Amici, R., Avanzi, N., Ballinari, D. et al. (2010) Optimization of 6,6-dimethyl pyrrolo[3,4-c]pyrazoles: identification of PHA-793887, a potent CDK inhibitor suitable for intravenous dosing. Bioorg. Med. Chem. 18, 1844–1853 10.1016/j.bmc.2010.01.042 20153204

[85] Abe, K., Yano, T., Tanno, M., Miki, T., Kuno, A., Sato, T. et al. (2019) mTORC1 inhibition attenuates necroptosis through RIP1 inhibition-mediated TFEB activation. Biochim. Biophys. Acta Mol. Basis Dis. 1865, 165552 10.1016/j.bbadis.2019.165552 31499159

[86] Wu, W., Wang, X., Sun, Y., Berleth, N., Deitersen, J., Schlutermann, D. et al. (2021) TNF-induced necroptosis initiates early autophagy events via RIPK3-dependent AMPK activation, but inhibits late autophagy. Autophagy 17, 3992–4009 10.1080/15548627.2021.1899667 33779513 PMC8726653

[87] Xie, Y., Zhao, Y., Shi, L., Li, W., Chen, K., Li, M. et al. (2020) Gut epithelial TSC1/mTOR controls RIPK3-dependent necroptosis in intestinal inflammation and cancer. J. Clin. Invest. 130, 2111–2128 10.1172/JCI133264 31961824 PMC7108921

[88] Liu, Q., Qiu, J., Liang, M., Golinski, J., van Leyen, K., Jung, J.E. et al. (2014) Akt and mTOR mediate programmed necrosis in neurons. Cell Death Dis. 5, e1084 10.1038/cddis.2014.69 24577082 PMC3944276

[89] Wang, Z., Feng, J., Yu, J. and Chen, G. (2019) FKBP12 mediates necroptosis by initiating RIPK1-RIPK3-MLKL signal transduction in response to TNF receptor 1 ligation. J. Cell Sci. 132, jcs227777 10.1242/jcs.227777 31028177

[90] Yang, Z.H., Wu, X.N., He, P., Wang, X., Wu, J., Ai, T. et al. (2020) A non-canonical PDK1-RSK signal diminishes Pro-caspase-8-mediated necroptosis blockade. Mol. Cell 80, 296–310.e6. 10.1016/j.molcel.2020.09.004 32979304

[91] Frank, D., Vaux, D.L., Murphy, J.M., Vince, J.E. and Lindqvist, L.M. (2019) Activated MLKL attenuates autophagy following its translocation to intracellular membranes. J. Cell Sci. 132, jcs220996 10.1242/jcs.220996 30709919

[92] Bach, M., Larance, M., James, D.E. and Ramm, G. (2011) The serine/threonine kinase ULK1 is a target of multiple phosphorylation events. Biochem. J. 440, 283–291 10.1042/BJ20101894 21819378

[93] Egan, D.F., Shackelford, D.B., Mihaylova, M.M., Gelino, S., Kohnz, R.A., Mair, W. et al. (2011) Phosphorylation of ULK1 (hATG1) by AMP-activated protein kinase connects energy sensing to mitophagy. Science 331, 456–461 10.1126/science.1196371 21205641 PMC3030664

[94] Kim, J., Kundu, M., Viollet, B. and Guan, K.L. (2011) AMPK and mTOR regulate autophagy through direct phosphorylation of Ulk1. Nat. Cell Biol. 13, 132–141 10.1038/ncb2152 21258367 PMC3987946

[95] Newton, K. and Manning, G. (2016) Necroptosis and inflammation. Annu. Rev. Biochem. 85, 743–763 10.1146/annurev-biochem-060815-014830 26865533

[96] Dondelinger, Y., Hulpiau, P., Saeys, Y., Bertrand, M.J.M. and Vandenabeele, P. (2016) An evolutionary perspective on the necroptotic pathway. Trends Cell Biol. 26, 721–732 10.1016/j.tcb.2016.06.004 27368376

[97] Bossen, C., Ingold, K., Tardivel, A., Bodmer, J.L., Gaide, O., Hertig, S. et al. (2006) Interactions of tumor necrosis factor (TNF) and TNF receptor family members in the mouse and human. J. Biol. Chem. 281, 13964–13971 10.1074/jbc.M601553200 16547002

[98] Jacobsen, A.V. and Murphy, J.M. (2022) CRISPR deletions in cell lines for reconstitution studies of pseudokinase function. Methods Enzymol. 667, 229–273 10.1016/bs.mie.2022.03.054 35525543

[99] Tanzer, M.C., Khan, N., Rickard, J.A., Etemadi, N., Lalaoui, N., Spall, S.K. et al. (2017) Combination of IAP antagonist and IFNγ activates novel caspase-10- and RIPK1-dependent cell death pathways. Cell Death Differ. 24, 481–491 10.1038/cdd.2016.147 28106882 PMC5344208

