# Supplementary material for: Inhibitors identify an auxiliary role for mTOR signalling in necroptosis execution downstream of MLKL activation
Source: Biochem J. 2024 Aug 27;481(17):1125–42. doi: 10.1042/BCJ20240255 (PMC11555701; doi:10.1042/BCJ20240255)

Inhibitors identify an auxiliary role for mTOR signalling in necroptosis execution downstream of MLKL activation

Sarah E. Garnish[1,2,*,†], Christopher R. Horne[1,2,3], Yanxiang Meng[1,2], Samuel N. Young[1], Annette V. Jacobsen[1,2], Joanne M. Hildebrand[1,2] and James M. Murphy[1,2,3*]

[1] Walter and Eliza Hall Institute of Medical Research, 1G Royal Parade, Parkville, VIC 3052, Australia

[2] Department of Medical Biology, University of Melbourne, Parkville, VIC 3052, Australia

[3] Drug Discovery Biology, Monash Institute of Pharmaceutical Sciences, Monash University, Parkville, VIC 3052, Australia

* To whom correspondence may be addressed sarah.garnish@monash.edu and jamesm@wehi.edu.au

[†] Current address: Monash Biomedicine Discovery Institute, Department of Microbiology, Monash University, Clayton, Victoria, 3800

Keywords: RIPK1, RIPK3, mTORC1, MLKL, cell death

Supplementary Figure 1. GSK′872 inhibits MLKL WT executed necroptosis at high concentrations. A - B *MLKL*$^{-/-}$ and *MLKL*$^{-/-}$ *RIPK3*$^{-/-}$ HT29 cells stably transduced with *MLKL*^{WT} or *MLKL*^{R30E} were treated with doxycycline (Dox) alone or in combination with TSI (TNF, Smac mimetic, IDN-6556), in the presence of RIPK3 kinase inhibitor, GSK′872, at increasing concentrations. IncuCyte SX5 imaging was used to quantify the percentage of cell death by determining the number SYTOX Green-positive cells (dead cells) relative to the number of DRAQ5-positive cells (total cell number) at 48 h. Data are plotted as mean ± SEM of *n* = 3. Two independent cell lines transduced with *MLKL*^{R30E} were generated; one was assayed in *n* = 1 and the other in *n* = 2, for a total of *n* = 3, independent experiments.

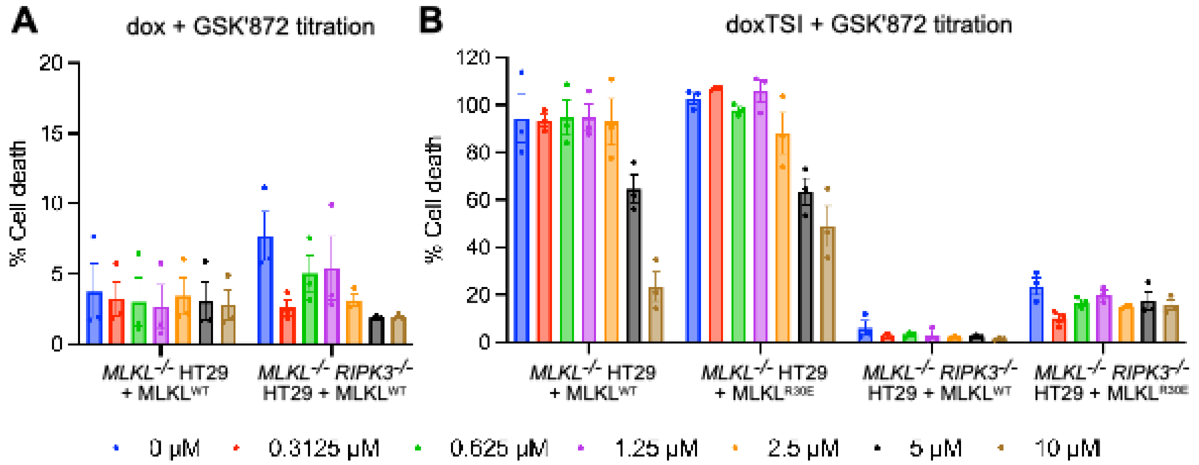

Supplementary Figure 2. Positive compound hits exhibit inhibition at different concentrations.

A – N Evaluation of R30E MLKL constitutive death inhibition at varying compound concentrations. **(A – M)** *MLKL*$^{-/-}$ HT29 cells were stimulated with 100 ng/mL doxycycline to induce expression of MLKL R30E and stimulated concurrently with compound at 0.25 – 4 µM. **(N)** *MLKL*$^{-/-}$ HT29 cells were co-stimulated with 100 ng/mL doxycycline and PHA-793887, Ridaforolimus, Temsirolimus, Rapamycin, or ABT-578 at 25, 50 or 100 nM. **(O)** *MLKL*$^{-/-}$*, RIPK3*$^{-/-}$ HT29 cells were stimulated simultaneously with 1 µM of compound and 100 ng/mL doxycycline to induce MLKL R30E expression. **(A – O)** Cell death was quantified at 44 hours as percentage by determining the number SYTOX Green-positive cells (dead cells) relative to the number of DRAQ5-positive cells (total cell number) using IncuCyte SX5 live cell imaging. Cells were assayed in $n = 3$ **(G, N, O)**, $n = 4$ **(H, I, M)**, $n = 5$ **(A, B, C, D, F, K, L)** or $n = 6$ **(E, J)** independent experiments with data plotted as mean ± SEM. *P* value calculated using an unpaired, two-tailed Students t-test. *p<0.05, **p<0.01, ***p<0.001

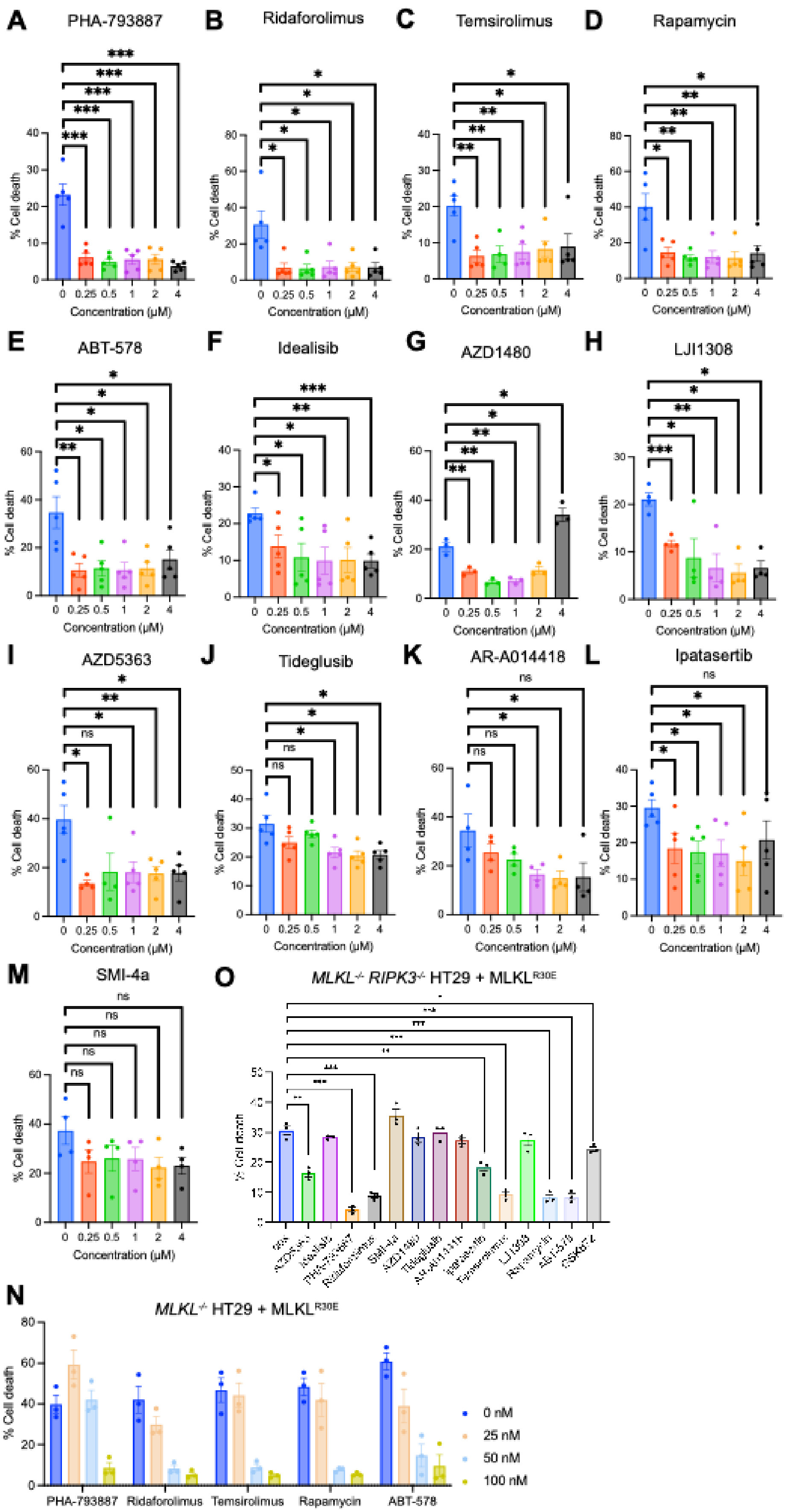

A
PHA-793887
B
Ridaforolimus
C
Temsirolimus
D
Rapamycin
E
ABT-578
F
Idealisib
G
AZD1480
H
LJI1308
I
AZD5363
J
Tideglusib
K
AR-A014418
L
Ipatasertib
M
SMI-4a
O
MLKL-/- RIPK3-/- HT29 + MLKL R30E
N
MLKL-/- HT29 + MLKL R30E
% Cell death
Concentration (μM)
0 nM
25 nM
50 nM
100 nM
PHA-793887
Ridaforolimus
Temsirolimus
Rapamycin
ABT-578

Supplementary Table 1. 429 screened kinase inhibitors

Compound	Fold decrease in % cell death
Chloroquine phosphate	-0.0405249
GSK621	-0.0060663
xMD8-92	0.24858143
GSK2636771	0.06102663
AMG337	-0.4311096
ETC-1002	0.04173598
PRT062607 (P505-15_BIlB057) Hcl	0.07542861
GSK2292767	0.21820026
LY2228820	-0.0468782
7_8-dihydroxyflavone	-0.3046597
GSC-0349	0.40044252
Tyrphostin AG 1296	-0.0939585
PQ 401	-0.4659934
Cobimetinib	-1.0609905
Butein	0.0553905
Olmutinib	-0.2244427
SMI-4a	0.24885357
AZD3759	-0.2009471
ASP3026	-0.0039963
PLX7904	-0.0106042
Entrectinib	-0.38863
CC-223	0.60982089
TCS 359	0.27914002
Mitefosine	-0.204019
Pacritinib	-1.9440588
Palbociclib	-0.120877
LY3023414	-0.1827803
VPS34-IN1	-0.462318
VE-821	-0.622079
AZD5363	0.54575239
APTSTAT3-9R	-0.3840816
Oclacitinib	0.21073002
P276-00	0.13713979
Prerifosine	-0.1192926
ETP-46464	-0.3244138
GSK2292767	0.14275473
Dorsomorphin 2HCl	-1.4559273
Oimutinib	-0.091818
Mitefosine	-0.0398052
ONO-4059	-0.0139509
AZD2858	-0.9997674

PP1	0.13339578
GNE-9605	0.02412315
SGI-7079	-0.8281839
LFM-A13	-0.0457122
JNK inhibitor IX	0.16872134
LDN-214117	-0.2912278
MLN2480	-0.5729607
SSR128129E	-0.0910189
PD173955	-0.811333
GNF-5	-0.2567442
CC-292	-0.708888
RN486	-0.0433536
Certinib	-0.5518132
Osimertinib	-0.1975361
FIIN-2	0.22636921
CGI1746	-0.6019664
PF-431396	-0.0176005
G-749	-0.2868299
DDR1-IN-1	-0.052458
ANA-12	-0.0723448
CNX-774	0.07325858
CNX-2006	-0.0569623
Rociletinib	-0.1205487
WH-4-023	-0.0774249
AZD3463	0.0773221
PF-3758309	-0.2568879
abemaciclib	-0.2032947
SU9516	0.05573734
Aktl-1/2	-0.1004359
D4476	-0.0073294
VE-822	-0.0797677
URMC-099	-0.7460842
LJI308	0.25707804
Anacardic Acid	0.20765506
KN-62	0.01000502
IPA-3	0.05931088
MK-8745	-0.0205436
Purvalanol A	0.15712694
LY2090314	-0.188939
AMG319	0.02722871
AZD6738	0.2477438
BLZ945	-0.0144258
SC1	-0.5212712

10058-F4	0.08855985
TF009	0.02680453
IM-12	0.06143401
LJH685	0.08565436
Bikinin	0.03050886
Ro-3306	0.08129365
Ulixertinib	-0.390547
SU6656	-0.8620859
TIC10	0.01842245
AZD1080	-0.0119338
AT13148	0.13175824
XMD8-92	-0.232065
Ro3280	-0.8234966
SKI II	-0.1893463
Losmapimod	-0.0505795
GNE-0877	-0.6233195
Sorafenib	-0.2853192
HS-173	-0.5947267
CGK 733	0.21057786
KN-93 phosphate	-0.1918577
Ehop-016	-0.6734572
FRAX597	-0.482735
Urprosertib	0.1849065
Skepinone-L	0.10665542
PF_543	-0.0933528
WZ4003	-0.6960127
Bay 11-7085	0.20520488
GSK2334470	0.0068028
ERK5-IN-1	-0.7624297
Tepotinib	0.10167927
CX-6258 Hcl	-0.1801118
SB239063	0.16655392
AZD1208	0.16216467
Zotarolimus (ABT-578)	0.32053149
HTH-01-015	-0.2235406
RKI-1447	0.04201273
AZ20	0.30536356
Bisindoylmaleimide I	-0.7560681
Fingolimod HCL	-0.3911107
ZM 306416	0.02011049
AZD4547	-1.3672333
PD168393	-0.0388351
Golvatinib	-0.0749811

Crenolanib	-0.1666422
PF-04691502	-0.0407915
NVP-BVU972	0.15643816
ZM 336372	0.10239138
VS-5584	-0.3366192
GSK1838705A	0.12484658
Trametinib	-0.4750527
TAK-285	0.25125281
PP2	0.14244941
AG-1478	0.00033954
Ibrutinib	-0.1595404
Facomitinib	-0.4543782
ALK-IN-1	-0.0394632
GNF-2	-0.0823925
Dabrafenib	-0.5367798
KX2-391	-0.3868299
ZM 323881	0.02420811
AS-604850	0.11326876
Milciclib	-0.4253718
Binimetinib	-0.2592474
JNK-IN-8	-0.0921086
TAE226	0.07951178
AMG-458	-0.0377978
Torin 2	0.12190223
PF-00562271	0.16561367
Dovitinib	-0.2326487
CHIR-98014	0.10220641
Cabozanitinib malate	0.15151577
TPCA-1	-0.236893
SC-514	-0.089076
Semaxanib	0.19168874
TAK-715	0.22346325
BI-D1870	0.03586908
PF-477736	-0.7352786
Varlitinib	0.43822617
Icotinib	0.07387357
WAY-600	-0.1781234
Tideglusib	0.26792124
IMD0354	-0.5333861
CEP-33779	0.03802728
PH-797804	0.15391477
VX-702	0.03903298
Dinaciclib	-0.0182165

S-Ruxolitinib	-0.0184934
SB415286	0.09443759
Flavopiridol Hcl	0.01913586
3-Methyladenine	0.10578959
A-769662	0.02037678
PHA-767491	0.24659652
Tyrphostin 9	-0.6758379
Ipatasertib	0.23257124
WP1066	0.31459776
R547	0.18082941
Duvelisib	0.48512327
Vistusertib	0.0344859
Tofacitinib	-0.0711437
GW5074	-0.3445876
TAK-901	-0.481742
Tofacitinib	-0.1123875
MK-5108	-0.0399101
Sotrastaurin	-0.8015436
CAY10505	-1.0901069
Fedratinib	-1.2711399
AMG-900	-2.4212745
PF-562281	0.26083329
Apitolisib	-0.3998758
CH5132799	-0.1420317
Go 6983	-0.6138957
WHI-P154	-0.0361985
SAR131675	-0.0420243
TF101209	-0.5352343
Tyrphostin AF 879	-1.8978113
CHIR-124	-1.4574208
CUDC-907	-1.5462262
Alpelisib	-0.0820406
INK 128	-0.3479076
TDZD-8	-0.3917955
CHIR-99021	-0.1930263
AZ628	-0.3723608
XL019	-0.0611435
Tivantinib	-0.1671339
BAY 11-7082	0.08907177
MK-2461	-0.8053556
NVP-BSK805 2HCL	-0.7576843
Pazopanib	-0.008006
Schisandrin B	0.13268614

CZC24832	-0.0492883
IKK-16	-0.7828093
Fasudil	-0.2683456
KU-60019	-0.4228989
AST-1306	-1.0498926
BMS-777607	-0.0710016
Pimasertib	-0.6412906
AT7867	-0.5093821
AZD8330	-1.2675819
R406	-0.3525537
KRN 633	0.04667716
CCT128930	0.21839026
Volasertib	-0.7851232
Doramapimod (BIRB 7960	0.33644795
SP600125	-0.4381002
H89 2HCL	-0.0908502
CP-673451	-0.1274372
AICAR	0.08580934
PD318088	-0.1414709
Ki8751	0.21710291
PF-573228	0.00478819
Quizartinib	-0.288081
Apatinib	-0.1004807
Genistein	-0.0572942
PF-4708671	0.2285693
AT7519	-0.4548543
Lapatinib	-0.3038144
OSI-420	0.04933999
R406	0.08560969
AS-252424	-0.369015
AZ960	-0.3554994
Asiatic Acid	-0.0288685
Thiazovivin	0.23971213
Hesperadin	-2.5470527
AZD8055	-0.3219834
Omipalisib	0.04308302
AZD5438	0.17957679
PHT-427	0.29123411
BMD-265246	0.20461036
Degrasyn	0.45064509
BS-181 Hcl	-0.2370265
A-674563	0.16655665
Tie2 kinase inhibitor	0.42396368

BIX 02189	0.03677388
Voxtalisib	-0.0366953
PHA-793887	0.28965955
KW-2449	-0.3265447
Phenformin HCl	-0.1711074
PIK-294	-0.1079404
PIK-93	-0.515252
SB590885	-0.0816097
Gandotinib	-0.6923412
LY2603618	-0.1519462
OSI-027	0.13370723
HMN-214	0.08893952
GSK461364	-0.9226705
BIX 02188	-0.0469689
PIK-93	-0.3562587
SB590885	-0.2408273
Gandotinib	0.01085854
AZD1480	0.25964876
Idealisib	0.27723881
GSK492286A	0.04071098
Torkinib	-0.3598426
AEE788	-0.0625116
Pelitinib	-0.3895753
PIK-293	0.4117966
Quercetin	0.08650997
TWS119	0.00559156
TAK-733	-0.2889716
Momelotinib	-0.008504
PHA-680632	-2.2366734
Daphnetin	0.14404944
Rigosertib	-0.4939113
AZD6482	0.37713686
SGI-1776	-0.3441689
Aurora A inhibitor I	-0.3417894
AZD7762	-0.2485455
Indirubin	-0.0453315
Buparlisib	0.10350126
PD0325901	-0.3120149
PF-04217903	0.02789165
Dasatinib	-0.9659683
PD98059	0.04810609
CP-724714	-0.1736308
Afatinib	0.05856454

CYC116	-0.1344925
YM201636	-0.3668497
AG-490	-0.0148836
Refametinib	-0.2226589
Vemurafenib	-0.6079179
GSK690693	0.09069869
BMS-536924	-0.8046526
Sunitinib Malate	-1.4139638
SNS-314 Mesylate	-0.3608054
Orantinib	0.02658796
Honokiol	0.02460899
MGCD-265	-0.8667051
WYE-125132	-0.5607472
RAF265	-0.5281368
Ponatinib	-0.8761699
BX-912	-0.3461851
A66	-0.0650637
Mubritinib	0.20577684
PP121	-0.3967916
BMS-794833	-0.0643706
Fostamatinib	-0.1608227
NVP-BHG712	-0.4974435
Telatinib	0.02550119
Chrysophanic Acid	-0.1330479
Rebastinib	-0.0588676
TGX-221	0.12422004
Bosutinib	-0.0204938
ENMD-2076	-1.5380366
MLN8054	-0.0249565
Bl2536	-0.6663597
Roscovitine	-0.0033107
CUDC-101	-0.6027135
CHIR-99021	-0.421929
PHA-665752	-0.4489671
JNJ-7706621	-0.042166
Barasertib	-0.1408329
BMS-754807	-0.3183567
Alisertib	-0.1103674
Brivanib Alaninate	-0.1882721
Flavopiridol	-0.3653941
KU-0063794	-0.1859941
Everolimus	0.25145067
PLX-4720	0.0823651

AG-1024	0.01344679
SB203580	-0.1631383
Triciribine	0.09456852
SNS-032	-0.5186033
WYE0354	0.23316279
LY294002	0.27415703
Y-27632	-0.0678873
Temsirolimus	0.22855436
BMS-540215	0.0567024
BMS-599626	-0.0922905
GDC-0941	-0.5156996
SL-327	-0.0996373
SB202190	0.00237347
PHA-739358	-0.7394955
SU11274	-0.6806088
OSU-03012	-0.7418398
SB216763	-0.4450388
Enzastaurin	-0.9312847
MK-2206	0.29925848
Crizotinib	0.01539736
ZM 447439	-0.3267052
U0126-EtOH	-0.1124054
AZD2171	-0.3275906
BIBF 1120	0.0684369
GDC-0879	-0.105675
Rapamycin	0.44045878
Ridaforolimus (Deforolimus_MK-8669)	0.43805374
Linisitinib	-0.0608465
ZSTK474	0.35841469
Tozasertib	-1.0658166
Nilotinib	-0.2263975
BX-795	-1.8839578
OSI-930	0.05806826
Lenvantinib	0.08492532
PD173074	-0.096109
BMS-907351	0.03320073
Dovitinib	-0.7440486
Axitinib	-1.7599929
PD184352	-0.1498909
Tivozanib	0.09892976
WZ8040	0.07589672
Gefitinib	0.07844606

GSK1904529A	-0.2467547
NVP-AEW541	0.06956223
BAY 7304506	0.11147687
KU-55933	-0.4206395
Linifanib	0.00933075
WZ4002	-0.3572975
JNJ-38877605	-0.30357
AMG-706	-0.2456215
GSK1363089	-1.0023105
AZD0530	-0.8367411
AB1010	-0.3132263
PI-103	0.07329861
GW786034 HCL	0.21363329
Vatalanib	-0.0199897
MP-470	-0.363878
AZD6244	-0.2827635
Sorafenib Tosylate	-0.0329636
WZ3146	-0.4650804
Imatinib Mesylate	0.14064144
GNE-7915	-0.2342394
NU6027	0.13216533
GSK650394	-2.0849086
Vacquinol-1	0.06281515
Piceatannol	0.00267276
AZD2932	-0.0356779
GW441756	-0.0022164
AZD8931	0.19159713
TG100-115	0.05037356
AT9283	-1.3717952
GW-572016	-0.0261566
CEP-32496	-0.0545657
ZM 39923	-0.0977243
NSC 23766	0.06176297
BMS-345541	0.14448733
DASA-58	-0.5943354
AG-18	-0.1193608
Bl-78D3	-0.3188474
Dorsomorphin 2HCl	-1.5524144
LDC000067	0.13271111
VX-509	0.19074856
GLPG0634	-0.0526067
TAK-632	-0.0484299
GZD824	-0.5746653

AR-A011418	0.26068044
VX-11e	-0.5339115

FIGURE 1C

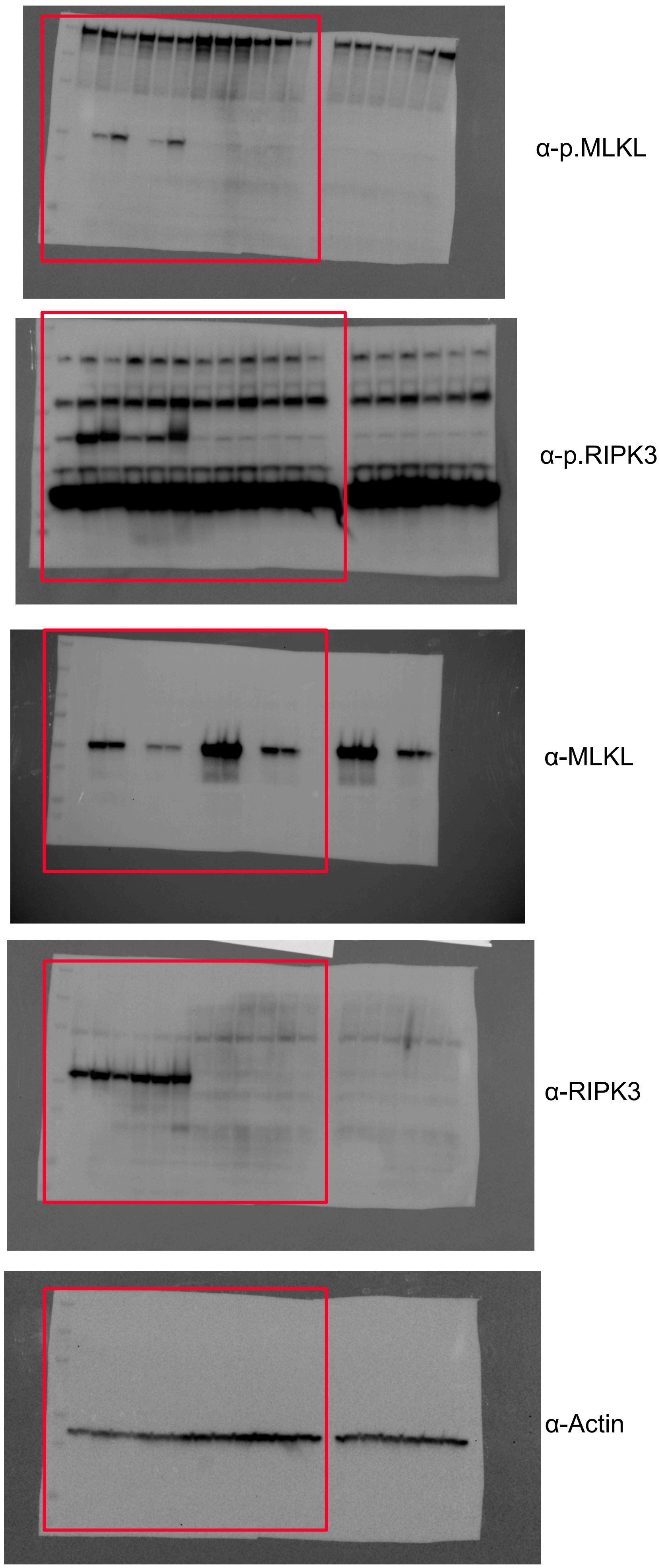

FIGURE 5

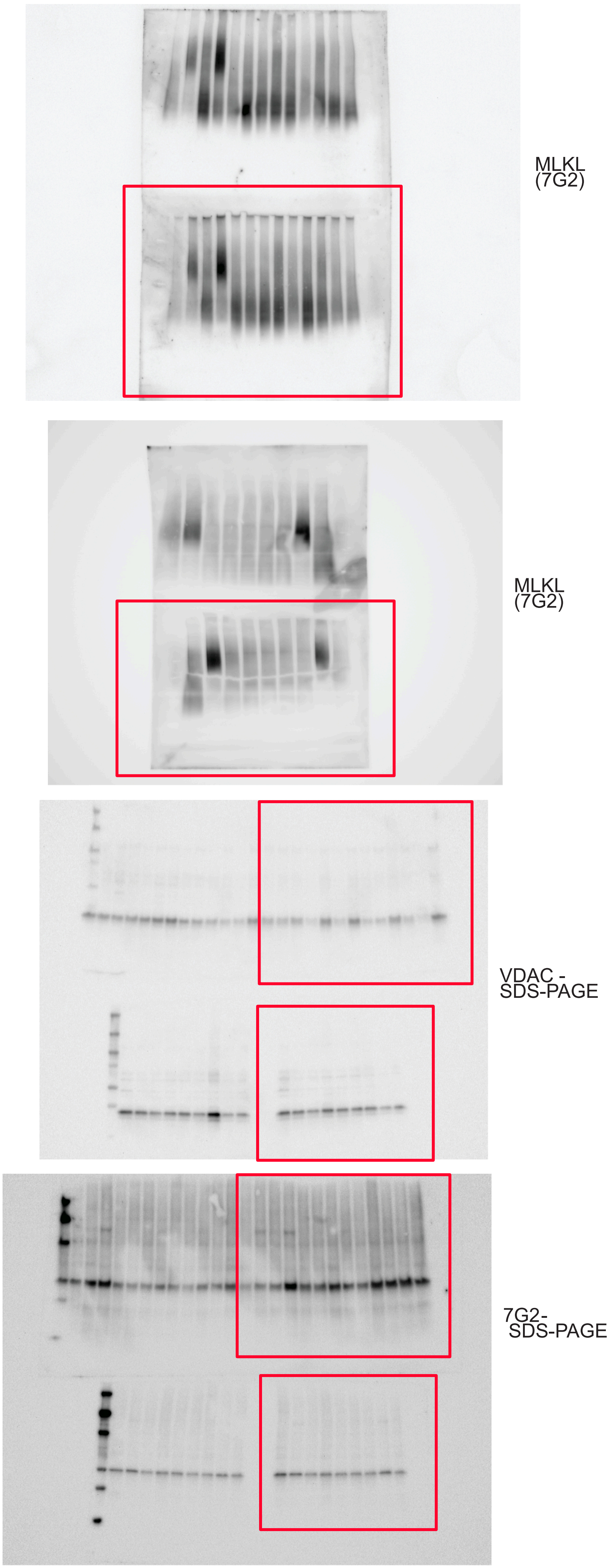

Supplement: Supplementary Material [file BCJ-481-1125-s1.pdf]
